# Supplementary material for: Comparing frequency of booster vaccination to prevent severe COVID-19 by risk group in the United States
Source: Nat Commun. 2024 Mar 6;15:1883. doi: 10.1038/s41467-024-45549-9 (PMC10917753; doi:10.1038/s41467-024-45549-9)
Supplement: Supplementary file 1 — Supplemental Information [file 41467_2024_45549_MOESM1_ESM.pdf]

Supplementary Materials

Comparing frequency of booster vaccination to prevent severe COVID-19 by risk group in the United States. *Nature Communications*. 2024.

Contents

Technical Appendix
- Simulating vaccine-induced and hybrid protection against COVID-19
  - Tables S1-S2: Summary of vaccine literature
  - Figures S1-S2: Protection and waning estimates
  - Figure S3: Relative vaccine effectiveness comparison
- Model calibration and validation
  - Figure S4: Time since last vaccine and/or infection
  - Table S3: Calibration performance
  - Figure S5: Calibration plots
  - Table S4: Validation performance
- Uncertainty analysis
  - Table S5: Model parameters for uncertainty analysis
- Sensitivity analyses
  - Figures S6-S7: Waning curves for sensitivity analyses
- Novel variant scenario analysis
  - Figures S8-S9: Waning curves for variant scenarios
- Dynamic model scenario analysis
  - Table S6: Social contact matrix
  - Table S7: Population characteristics in dynamic model
  - Table S8: Calibration performance

Appendix References

Supplemental Tables and Figures
- Table S9**:** Model prediction of risk of non-severe COVID-19.
- Tables S10-S11: Model prediction of risk of severe COVID-19 in immunocompromised.
- Table S12: Model prediction of risk of severe COVID-19 under no booster intervention.
- Tables S13-S14: Model prediction of risk of severe COVID-19 under different assumptions of waning.
- Tables S15-S16: Model prediction of risk of severe COVID-19 under different assumptions of vaccine effectiveness.
- Tables S17-S18: Model prediction of risk of severe COVID-19 under different assumptions of incidence.
- Tables S19-S21: Model prediction of risk of severe COVID-19 under different assumptions of baseline seroprevalence.
- Table S22: Model prediction of risk of severe COVID-19 under five-year-simulation.
- Table S23: Model prediction of risk of severe COVID-19 under delayed vaccination administration.
- Table S24: Model prediction of risk of severe COVID-19 under higher sub-clinical infection.
- Table S25: Model prediction of risk of severe COVID-19 under lower vaccine effectiveness for subsequent doses after the first dose.
- Figure S10: Plots of COVID-19 incidence over time.
- Figure S11: Risk of severe COVID-19 over time by baseline risk and waning protection.

Figure S12-S13: Variant scenario analysis, using two versions of immune evasion.
Figure S14-S15: Dynamic model analysis, using two assumptions on vaccine uptake.

Technical Appendix

In this appendix, we provide further methodologic description of the data, model structure, assumptions, and statistical analysis in this study.

Simulating vaccine-induced and hybrid protection against COVID-19

Literature to inform estimates on vaccine-induced and hybrid protection for severe and non-severe COVID-19

We used published studies on the level of protection against severe and non-severe COVID-19 generated from vaccination alone and hybrid immunity (vaccination and prior documented COVID-19) and the waning of this protection over time (Table S1-S2). We simulated vaccine-induced (without prior infection) and hybrid immunity separately given literature suggesting higher and more durable protection from hybrid immunity[1]. Waning was defined as changes in level of protection against severe and non-severe COVID-19 since the last vaccine dose or prior infection (whichever event was more recent), which is the definition mostly commonly employed in literature for this analysis.

Table S1: Summary of literature on vaccine-induced and hybrid protection against severe COVID-19.

Vaccine status	Prior infection	Follow up time	References
Absolute vaccine effectiveness estimates			
3-doses monovalent	Yes	8 months	Bobrovitz et al. (*Lancet ID, 2023)*[1]
		11 months	Carazo et al. (*Lancet Healthy Longevity, 2023)*[2]
		6 months	Lin et al. (*JAMA*, 2022)[a]
	No	8 months	Bobrovitz et al. (*Lancet ID, 2023)*[1]
		8 months	Ferdinands et al. (*BMJ, 2022)*[3]
Relative vaccine effectiveness estimates			
Bivalent booster dose	Yes	6 months	Lin et al. (*NEJM, 2023)*[4]
Bivalent booster dose	No	6 months	Lin et al. (*NEJM, 2023)*[4]

[a]This article was used as supportive evidence but did not provide absolute protection estimates.

Table S2: Summary of literature on vaccine-induced and hybrid protection against non-severe COVID-19.

Vaccine status	Prior infection	Follow up time	References
Absolute vaccine effectiveness estimates			
3-doses monovalent	Yes	8 months	Bobrovitz et al. (*Lancet ID, 2023)*[1]
		6 months	Andeweg et al. (*Nature Communications, 2022)*[5]
	No	8 months	Bobrovitz et al. (*Lancet ID, 2023)*[1]
		6 months	Andeweg et al. (*Nature Communications, 2022)*[5]

Generating estimates of protection and waning for severe and non-severe COVID-19

We simulated the impact of different booster vaccine schedules over a two-year time horizon. To do this, we needed data on the absolute protection against non-severe and severe COVID-19 for any person, specific to their vaccine status, prior infection status, and time since last vaccine and/or prior infection. Absolute protection was defined against an immune naïve individual. We used data available for both monovalent and bivalent COVID-19 vaccination (see Tables S1-S2) and performed statistical modeling on this data to estimate the waning of protection. For modeling protection against non-severe COVID-19, we used data from a meta-analysis that provided non-age-stratified estimates on protection and waning (Table S2)[1]. To create age-stratified estimates, we used age-stratified relationships from another study[5], which was represented in the meta-analysis. For modeling protection against severe COVID-19, we used data from persons with booster doses (3rd dose mRNA vaccine) by age group and prior infection status (Table S1). We used a linear mixed effects model calibrated to literature data on protective effectiveness and waning to generate estimates over a 24-month period. The model outcome was the log of 1 minus protective effectiveness against severe or non-severe COVID-19, with predictor variables of the log of months since last vaccine dose or COVID-19 illness (whichever was more recent), age group (18-49 years, 50-64 years, 65+ years), and prior infection status. Severe and non-severe COVID-19 were modeled separately. We estimated the mean, lower bound, and upper bound simultaneously by treating them as an ordinal variable (lower bound, mean, upper bound), calibrating to literature data using the mean and 95% confidence interval (for lower and upper bound) of protective effectiveness. For the linear mixed effects model, we included a random effect for each study. We included model weights to account for different sample size and level of precision in literature estimates; we defined the weight for each study as the inverse of the width of each estimate's 95% confidence interval. We used the R package 'lmer'. For the mild immunocompromised population, we assumed that protection was on average 13% lower than the immunocompetent population based on literature estimates[3], and adjusted waning curves accordingly. For the moderate/severe immunocompromised population, we assumed that waning of protection was on average 25% lower than the immunocompetent population, so we shifted the waning curves down 13% and increased rate of waning by 12%[3,6]. The estimates on protection against severe COVID-19 for vaccine alone and hybrid immunity are shown in Figure S1. The estimates on protection against non-severe COVID-19 for vaccine alone and hybrid immunity are shown in Figure S2. These estimates of protection against severe

COVID-19 aligned with recent data on the relative vaccine effectiveness of the bivalent booster (see Figure S3).

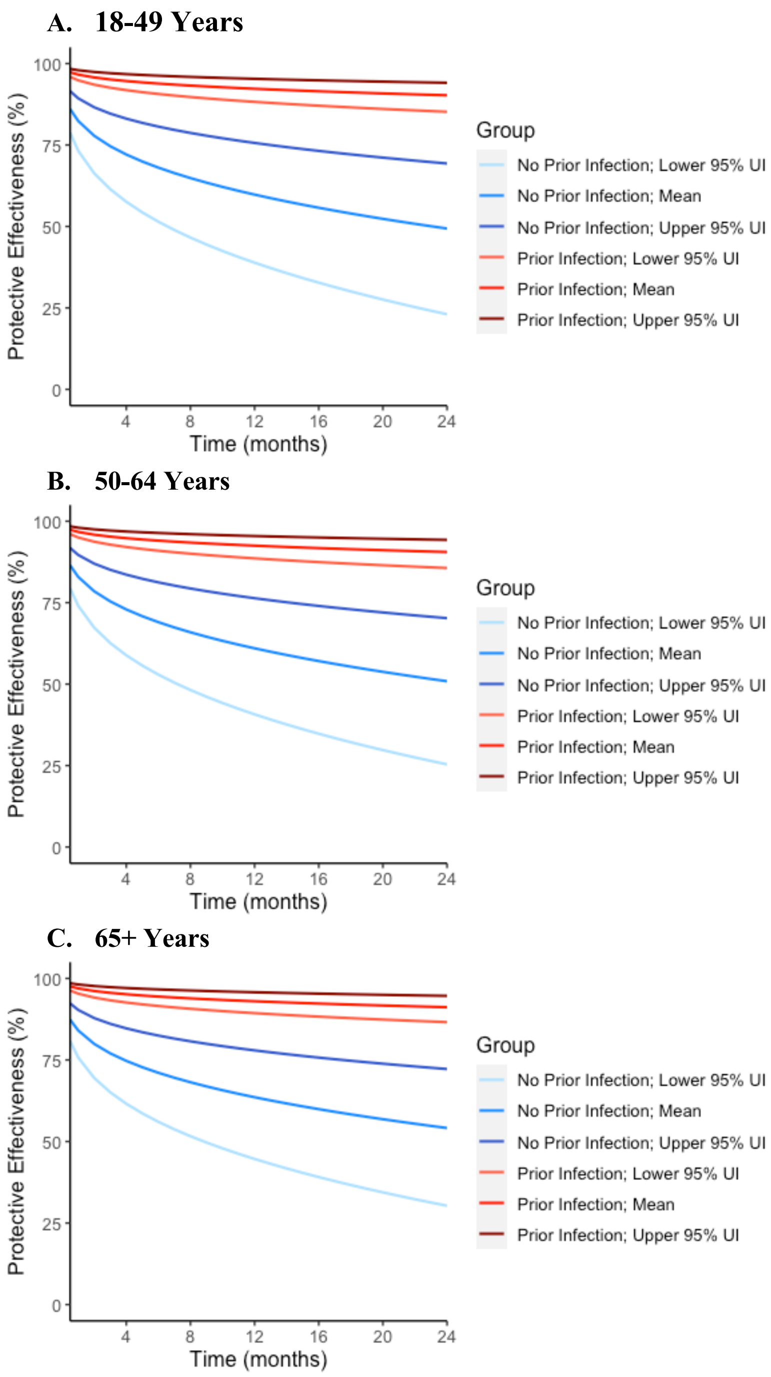

D. Immunocompromised (Mild)

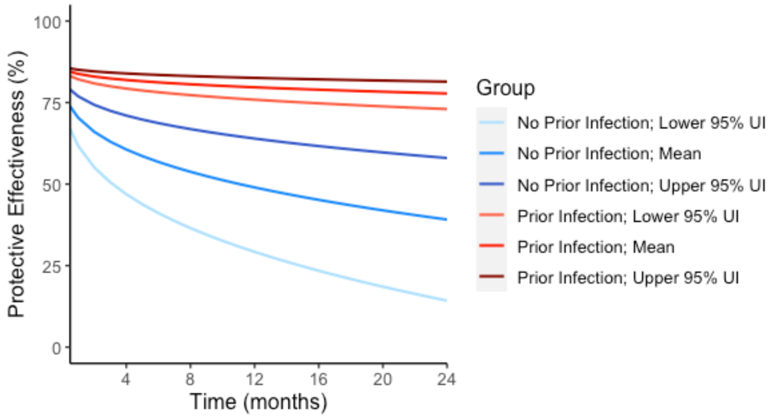

E. Immunocompromised (Moderate/Severe)

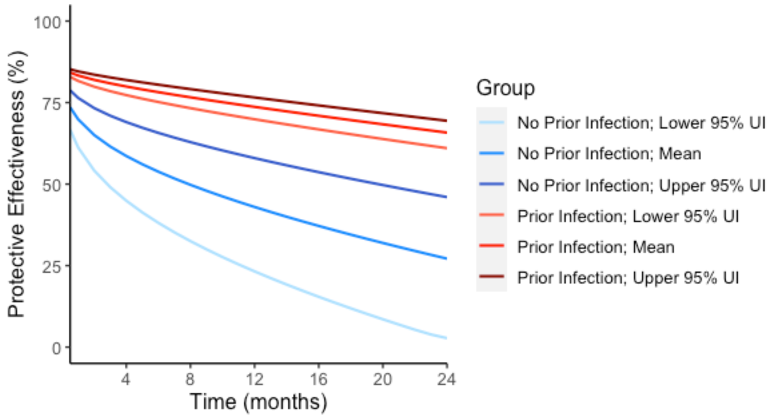

Figure S1: Protective effectiveness against severe COVID-19 generated from vaccine-induced and hybrid immunity by age group or immunocompromised status. Applying published literature, we estimated the absolute protection against severe COVID-19 from vaccine alone (blue line) and hybrid immunity defined by vaccination and prior infection (red line). We plot estimates for five risk groups: (A) 18-49 years; (B) 50-64 years; (C) 65+ years; (D) mild immunocompromised population; and (E) moderate/severe immunocompromised population. In the microsimulation, we use age-specific protection estimates for each immunocompromised population; these plots (D-E) are age-weighted curves for visualization purposes. We used a linear mixed effects model to calibrate to observed data, and then extrapolated these estimates over a 24-month time period. Each estimate included a 95% interval, based on the confidence intervals reported in the primary literature.

A. 18-49 Years

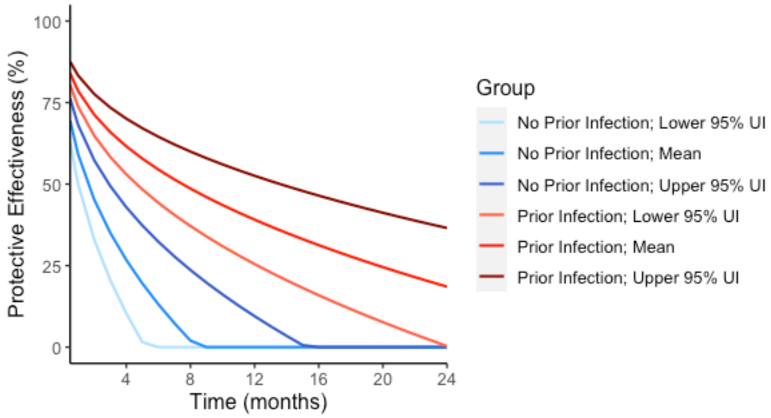

B. 50-64 Years

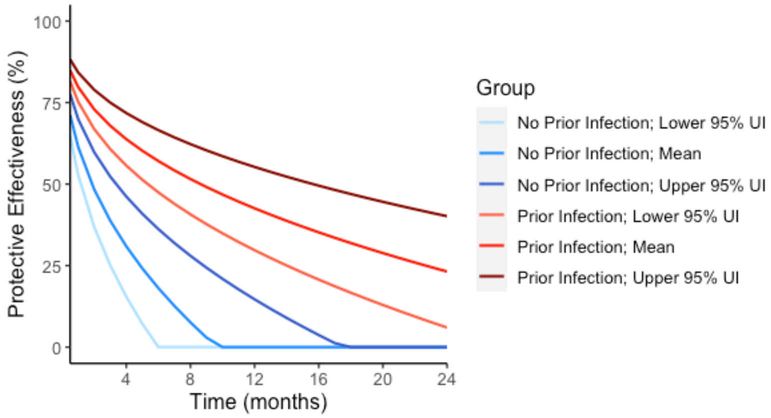

C. 65+ Years

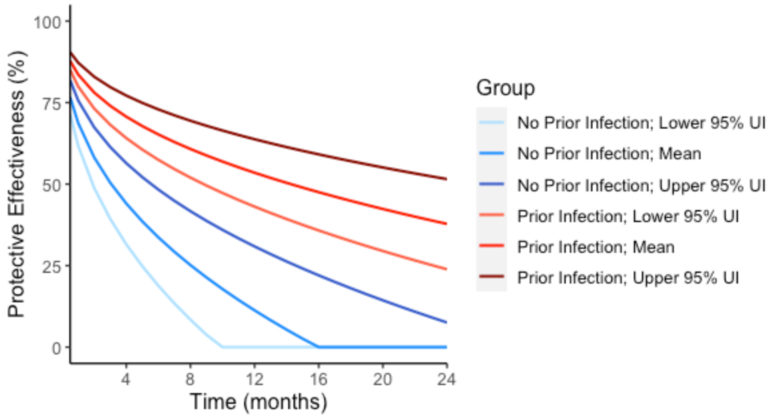

D. Immunocompromised (Mild)

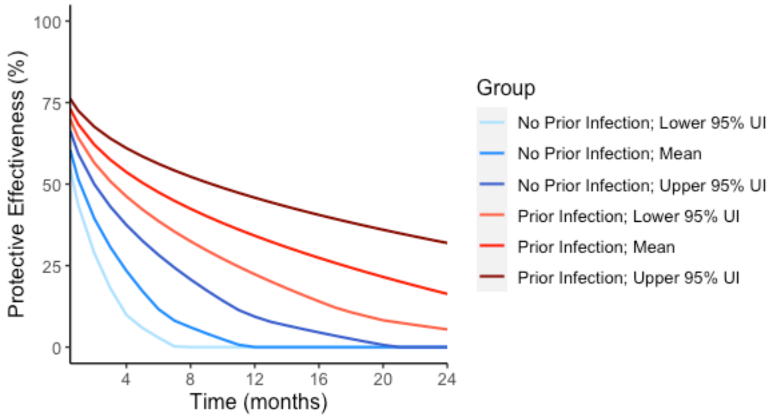

E. Immunocompromised (Moderate/Severe)

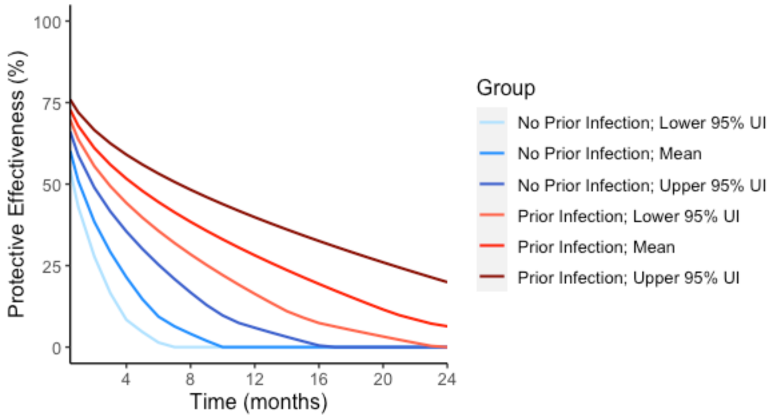

Figure S2: Protective effectiveness against non-severe COVID-19 generated from vaccine-induced and hybrid immunity by age group or immunocompromised status. Applying published literature, we estimated the absolute protection against non-severe COVID-19 (infection) from vaccine alone (blue line) and hybrid immunity defined by vaccination and prior infection (red line). We plot estimates for five risk groups: (A) 18-49 years; (B) 50-64 years; (C) 65+ years; (D) mild immunocompromised population; and (E) moderate/severe immunocompromised population. In the microsimulation, we use age-specific protection estimates for each immunocompromised population; these plots (D-E) are age-weighted curves for visualization purposes. We used a regression model to calibrate to observed data, and then extrapolated these estimates over a 24-month time period. Each estimate included a 95% interval, based on the confidence intervals reported in the primary literature.

Simulating protection from COVID-19 booster vaccination

We simulated the benefit of a booster dose to reverse waning of protection and restore the maximal protection against severe and non-severe COVID-19 (see Figures S1 and S2). As an example, for the outcome of severe COVID-19, for a person who is 65+ years (Figure S1, panel C), vaccinated but not previously infected, and last vaccinated or infected 4 months ago, they have a protective effectiveness of about 75% against severe COVID-19, compared to an immune naïve person. If this person receives a booster dose, we model this as shifting them left on this waning curve to time=0, which is about 87% protection, after which they will wane back down the curve over time. Therefore, the impact of additional vaccination conservatively did not increase the absolute protective effectiveness previously achieved, but only restored the lost protection due to waning. This approach to vaccine modeling achieves relative vaccine effectiveness estimates for vaccine alone and hybrid immunity similar to published estimates on the mRNA booster, including monovalent and bivalent doses (Figure S3). While we simulated

bivalent mRNA doses, it may be reasonable to extrapolate this estimated protection to future COVID-19 booster vaccines such as a monovalent booster vaccine targeting XBB.1.5 or future vaccine-targeted variants, especially while longer term follow up data is not yet available. This model can be revised with updated vaccine data to inform decisions on new vaccine formulations as data becomes available. Ultimately, this approach will be relevant to new COVID-19 vaccine formulations if the generated protection is similar to the data and assumptions used in the study.

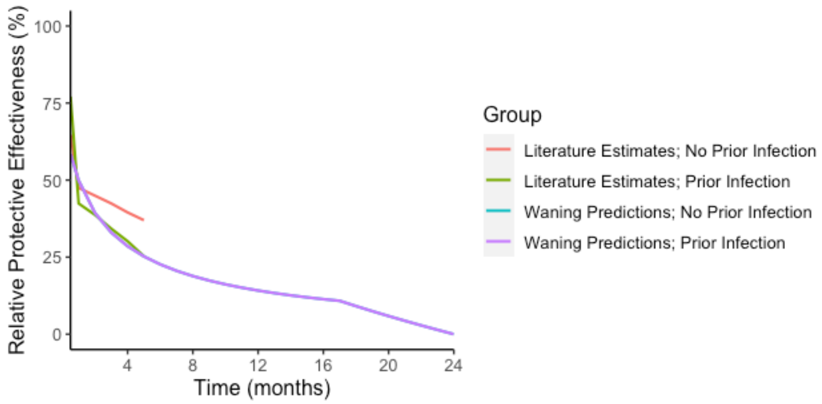

Figure S3: Relative vaccine effectiveness of a bivalent COVID-19 booster vaccine dose from literature[7] compared to estimate in the simulation.

Prior infection and serosurveillance data

Prior infection status for each person in the model was informed by age-specific seroprevalence estimates, which were obtained from US CDC estimates of serologic surveys with the nucleocapsid antibody (suggesting prior infection, not vaccine-induced antibody response).[8] The last survey date was from February 2022, thus age-specific seroprevalence estimates were updated as of this date. The model initialization for this study was September 2022, so we used cumulative COVID-19 case counts from March 2022 – August 2022[9] to adjust these age-specific seroprevalence estimates to account for this missing time period. We assumed a 2x multiplier to account for case underreporting in the general population. For the immunocompromised population, we created a seroprevalence estimate based on their corresponding age group[10]. Assigning a time since last infection is further described in “Estimation of time since last COVID-19 vaccine or illness” section below.

Model calibration

Estimation of time since last COVID-19 vaccine or illness

For calibration of the model (initializing the model to approximately September 2022), we estimated the level of protection against severe and non-severe COVID-19 for each person in the model. This protection was specific to their vaccine status, prior infection status, and time since last vaccination or infection (to account for waning of protection). To simulate time since last COVID-19 vaccine or infection for each person, we separately simulated distributions of time since vaccination and COVID-19 using publicly available data. We calculated time since last COVID-19 event by taking the most recent time among the time since last vaccination, time since infection, or time since reinfection (see Figure S4, Panel D). The ‘time-since’ distributions by age-group are shown in Figure S4.

For time since last vaccination, we used publicly available data from California COVID-19 vaccine administration of monthly monovalent booster dose counts over time (up until September 1, 2022), which is likely broadly representative of the United States[11]. We simulated the most recent vaccine dose by randomly sampling from this distribution. This timeframe was chosen to follow the period prior to bivalent booster introduction. We assumed that the time since last booster dose was conditional on the number of monovalent booster doses received by each person (either 1 or 2 booster doses); (see Figure S4, Panel A). We used available data to assign each individual either 1 or 2 monovalent booster doses (with the exception of the 18-49 year age group which we assumed were all 1 booster dose)[1]. If a person received multiple booster doses, we sampled from a more recent time period.

For time since last infection (if applicable), we simulated their time since last COVID-19 infection by randomly sampling from publicly available data on the distribution of monthly COVID-19 cases over time (see Figure S3, Panel B)[13]. We assumed 10% were reinfection over this period (see Figure S4, Panel C).

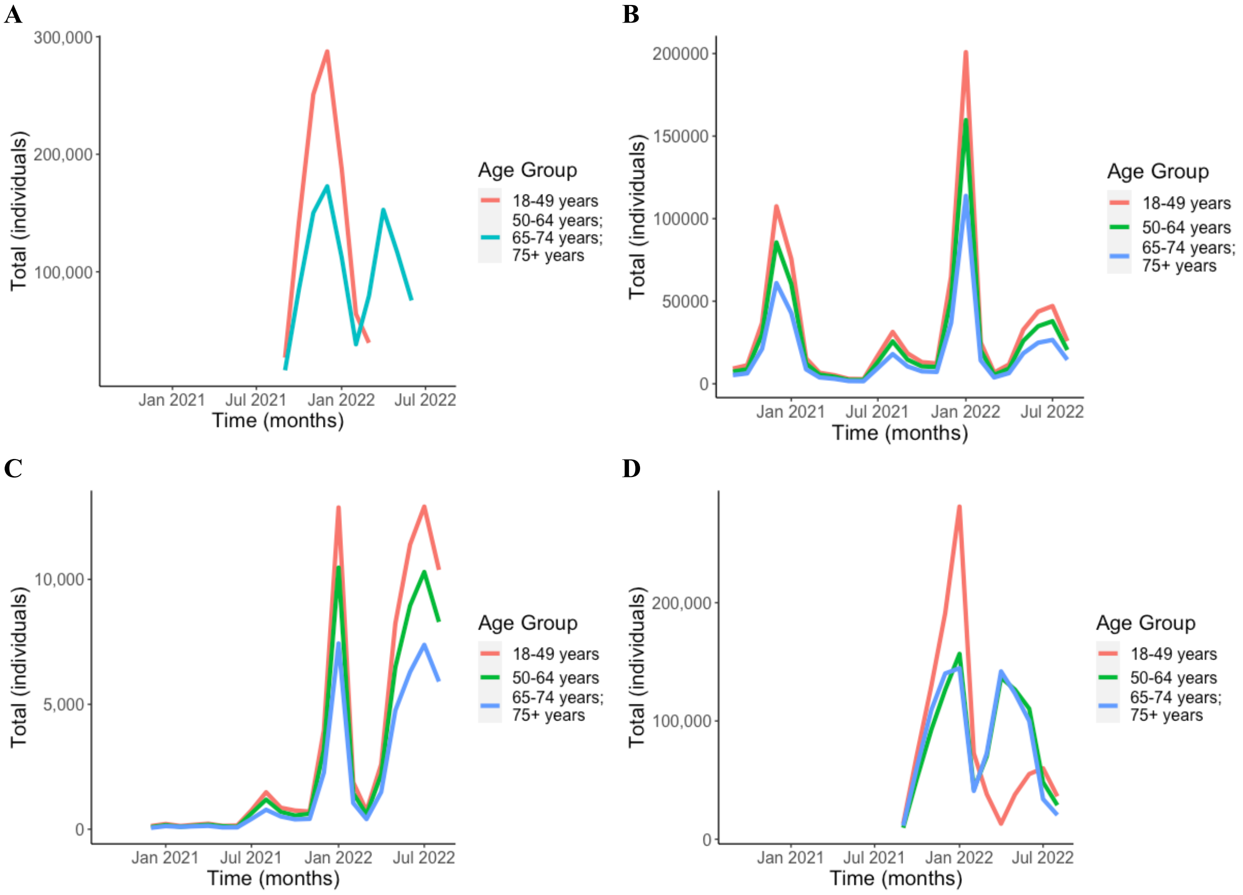

Figure S4: Data on COVID-19 vaccination, clinical cases, and re-infection to inform time since last COVID-19 vaccine or illness in the simulated population. We used publicly available data from California on: (A) COVID-19 monovalent booster dose administration; (B) COVID-19 clinical cases; (C) COVID-19 reinfections, assuming 10% reinfection. We sampled from these distributions to generate a distribution for time since last COVID-19 vaccine or infection (panel D).

Model calibration and prediction

The model was calibrated to observed data on age-specific estimates of monthly severe COVID-19 incidence (per 100,000 persons). We used publicly available data from the US CDC on severe COVID-19 incidence (based on hospitalization) by age group, using data over the 6-month period preceding bivalent vaccine roll out (March 2022 – August 2022)[14]. We assumed all COVID-19 deaths were linked to a hospitalization, so we defined severe COVID-19 cases as those leading to hospitalization or death. To estimate severe COVID-19 incidence in vaccinated persons only, we adjusted these age-specific estimates of severe COVID-19 risk by removing the contribution from unvaccinated persons. For this, we used data on COVID-19 death in unvaccinated and vaccinated populations by age[9], unvaccinated population counts by age[15], and population age distributions[16]. For the immunocompromised population, we applied a 2.8x multiplier to the age-specific estimates of severe COVID-19 incidence to account for overall higher severity given infection in this population[10].

The model calibration was done analytically at model initialization (t=0). The model equation for severe COVID-19 was: $Risk_{severe\ COVID} = \lambda * (1 - P_t)$. Risk was estimated as a monthly probability of severe COVID-19. The λ term was equal to monthly risk in a fully susceptible person. The P_t term was level of protection at time 't' and defined as absolute protective effectiveness against severe COVID-19, with waning since last vaccine or infection (see Figure S1). The observed group-specific risk estimates for severe COVID-19 are shown in Table S3. The calibration plots of the simulated severe and non-severe COVID-19 outcome counts over time without any additional booster doses are shown in Figure S5 (counterfactual scenario).

We simulated non-severe COVID-19 given its role in generating protection and our assumed 90-day perfect immunity period. The model equation was: $Risk_{non-severe\ COVID} = \lambda * (1 - P_{t,non-severe}) * m_k$. Risk was estimated as a monthly probability of non-severe COVID-19. The λ term was equal to risk in a fully susceptible person (force of infection term), which was adjusted for non-severe risk using age-specific case multipliers m_k. The $P_{t,non-severe}$ term is level of protection at time 't' and defined as absolute protective effectiveness against non-severe COVID-19, with waning since last vaccine or infection (see Figure S2). Table S3 includes the multipliers (m_k) atop severe COVID-19 to generate the number of non-severe infections in the risk groups. These multipliers were informed by US CDC estimates of age-stratified ratios of infections and hospitalizations/deaths[17], available literature, and accounting for underreporting of cases. To estimate non-severe COVID-19 in the immunocompromised group, we used an age-adjusted multiplier informed by the same CDC estimates of age-stratified ratios of infections, age distribution of the immunocompromised population[16,18], and cumulative risk ratio between immunocompromised and immunocompetent groups[10].

The full reporting of results for predicted severe COVID-19 outcomes under each booster schedule are in the main manuscript (Table 1) and Figure S10, and the results for predicted non-severe COVID-19 outcomes are shown in Table S9. The age-stratified results for predicted severe COVID-19 outcomes in immunocompromised groups are in Tables S10-S11. The results under no additional booster are in Table S12. We also included a set of risk estimates for severe COVID-19 based on baseline characteristics (age, immunocompromised status) and waning protection over time, which are shown in Figure S11.

Table S3: Model calibration with risk estimates for severe COVID-19.

Risk Group	Monthly Severe COVID-19 Risk (λ)[a]	Monthly Incidence, per 100,000 persons (Observed)	Monthly Incidence, per 100,000 persons (Model)	Non-Severe Infection Multiplier
18-49 years	0.00073<br>(0.00037 - 0.00238)	7.9	8.0<br>(7.9 - 8.8)	200
50-64 years	0.00111<br>(0.00060 - 0.00354)	15.8	16.5<br>(15.6 – 17.3)	79.6
65-74 years	0.00226<br>(0.00129 - 0.00568)	40.4	40.9<br>(40.3 – 42.3)	22.6
75+ years	0.00622<br>(0.00355 - 0.01565)	112.7	113.7<br>(112.6 – 117.2)	9.6
Immunocompromised (Mild)	0.00356<br>(0.00239 – 0.00575)	99.9	103.2<br>(101.3 – 106.3)	101.1
Immunocompromised (Moderate/Severe)	0.00320<br>(0.00221 – 0.00489)	99.9	103.4<br>(100.9 – 106.5)	101.1

Observed data from US CDC surveillance data on severe COVID-19 in the United States.
[a]The monthly severe COVID-19 risk estimate is multiplied by 1 minus the protective effectiveness in the model. We assumed mild and moderate/severe immunocompromised population had a similar risk of SARS-CoV-2 infection as the general population but 2.8x higher risk of severe disease given infection. These estimates are reported in this table as age-weighted averages.

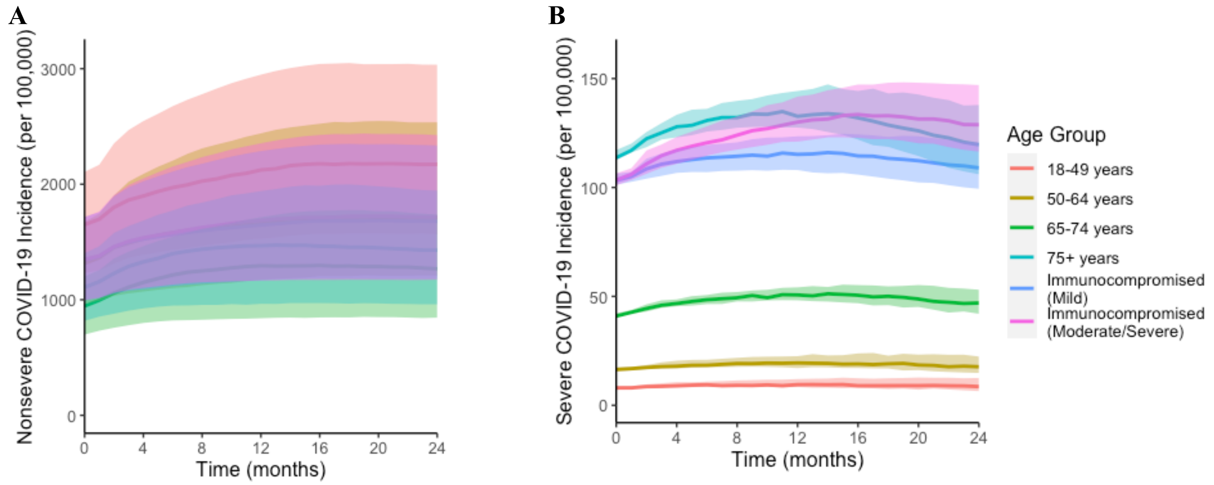

Figure S5: Monthly incidence of non-severe and severe COVID-19 outcomes in four age groups and two immunocompromised groups over a two-year simulation period with no additional COVID-19 booster vaccination. We used a microsimulation population of 1 million per risk group and estimated person-level protection against non-severe (panel A) and severe COVID-19 (panel B) based on each person's vaccine status, prior infection history, and time since last vaccine or natural infection. We modeled the waning of each person's pre-existing protection at the start of the simulation, as no additional COVID-19 booster vaccination was distributed. The uncertainty intervals are based on 95% CI of published literature of waning data, in addition to uncertainty characterized in the age-specific seroprevalence and non-severe infection multiplier estimates.

Model validation

For model validation, we performed a comparison of model-predicted outcomes and observed outcomes over the first 3 months of bivalent vaccination in the United States (September 2022 – November 2022). We obtained observed data on age-specific estimates of monthly severe COVID-19 incidence (per 100,000 persons) using the same US CDC dataset on severe COVID-19 incidence and analytical approach as previously described[14]. For the model-predicted outcomes, we simulated the one-time-dose booster schedule with the bivalent vaccine (coinciding with bivalent dose roll out), adjusting the number of vaccines that were released to match the proportions vaccinated with the bivalent booster by end of November 2022 in the United States[12,15,19]. We compared the average monthly incidence of severe COVID-19 outcomes over the 3-month period between the model prediction and observed data (Table S4).

Table S4: Model validation comparing model-predicted severe COVID-19 incidence and observed incidence over the first 3 months of bivalent vaccination in the United States (September 2022 – November 2022).

Risk Group	Severe COVID-19 Monthly Incidence, per 100,000 persons (Observed)	Severe COVID-19 Monthly Incidence, per 100,000 persons (Model)	Proportion Vaccinated with Bivalent Booster by November 2022 [a]
18-49 years	6.3	8.2	0.147
50-64 years	17.1	16.7	0.147
65-74 years	47.3	40.6	0.326
75+ years	141.9	111.2	0.326
Immunocompromised (Mild)	118.3	102.1	0.36
Immunocompromised (Moderate/Severe)	118.3	102.2	0.36

[a] The proportion vaccinated for the 18-49 and 50-64 years age groups are from 18+ years estimate. The proportion vaccinated used for the 65-74 and 75+ years age groups are for the 65+ years estimate.
We assumed mild and moderate/severe immunocompromised population had the same incidence of severe COVID-19 given limited reported data. In these populations, we assumed a similar risk of SARS-CoV-2 infection as the general population but 2.8x higher risk of severe disease given infection. These estimates are reported in this table as age-weighted averages.

Uncertainty analysis

To quantify uncertainty in the study findings, we generated uncertainty intervals (UI) for our model estimate that account for parameter uncertainty in model inputs. These intervals account for the uncertainty in protective effectiveness and waning over time, in addition to the uncertainty in baseline age-specific seroprevalence estimates and age-specific non-severe infection multipliers. For each model parameter, we created 'upper', 'mean', and 'lower' bound versions (Table S5) and ran simulations under each combination of model parameter bounds. The uncertainty interval represents the full bounds of the study outcomes under these parameter

combinations. The lower, mean, and upper bounds of the waning curves were generated from the 95% confidence intervals of the absolute protective effectiveness literature estimates (Tables S1-S2). The predicted estimates of waning protective effectiveness by age group and prior infection status are shown in Figures S1-S2. For baseline age-specific seroprevalence estimates, we set the lower bound to be 10% lower (in absolute terms) than the base case estimates and the upper bound to be 25% higher (in absolute terms) than the base case seroprevalence estimates. For the age-specific non-severe infection multipliers, we set the lower bound to be a 25% reduction and the upper bound to be a 25% increase from the base case non-severe infection multiplier estimates. We ran 25 simulations of each parameter set which achieved stable estimates. The model parameter ranges to generate the uncertainty interval are summarized in Table S5.

Table S5: Model parameters for characterizing uncertainty.

Model Parameter	Lower Bound	Mean	Upper Bound
**Waning Curves (severe and non-severe)**	Lower bound of waning curves	Mean waning curves	Upper bound of waning curves
**Baseline Seroprevalence**			
18-49 years	0.7237	0.8238	1
50-64 years	0.5579	0.6579	0.9079
65-74 years	0.3681	0.4681	0.7181
75+ years	0.3681	0.4681	0.7181
**Non-severe Infection Multipliers**			
18-49 years	150	200	250
50-64 years	59.7	79.6	99.5
65-74 years	16.95	22.6	28.25
75+ years	7.2	9.6	12

Sensitivity analysis

Pessimistic and optimistic waning

We conducted a sensitivity analysis modeling pessimistic and optimistic waning of protection against severe COVID-19 outcomes. We reduced the rate of waning (optimistic assumption) or increased the rate of waning (pessimistic assumption) by 10%, as shown in Figure S6. This was done separately for each risk group. Then we simulated the COVID-19 booster schedules in each risk group cohort. The predicted annual risk of severe COVID-19 outcomes under each booster schedule are in the main manuscript (Figure 3). The predicted severe COVID-19 outcomes under each booster schedule are in Tables S13 and S14.

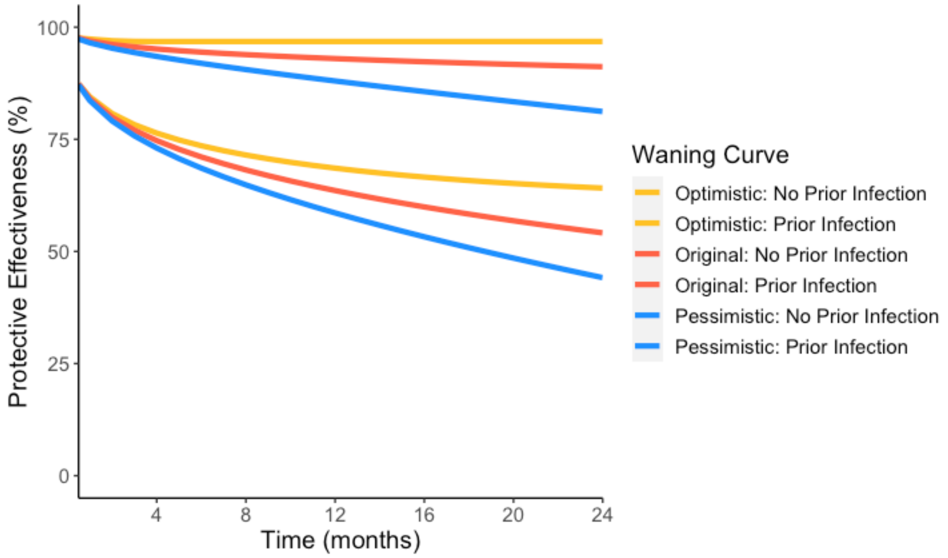

Figure S6: Protective effectiveness against severe COVID-19 generated from vaccine-induced and hybrid immunity with assumptions of pessimistic and optimistic waning. After generating the absolute predictions for vaccine-induced and hybrid immunity (red line), we reduced the rate of waning (optimistic assumption; yellow line) or increased the rate of waning (pessimistic assumption; blue line) by 10%.

Pessimistic and optimistic vaccine effectiveness

We conducted a sensitivity analysis modeling pessimistic and optimistic assumptions of overall protection against severe COVID-19 outcomes. We shifted the protection curves up (optimistic assumption) or down (pessimistic assumption) by 10%, as shown in Figure S7. Then we simulated the three COVID-19 booster schedules in each risk group cohort. The predicted annual risk of severe COVID-19 outcomes under each booster schedule are in the main manuscript (Figure 3). The predicted severe COVID-19 outcomes under each booster schedule are in Tables S15 and S16.

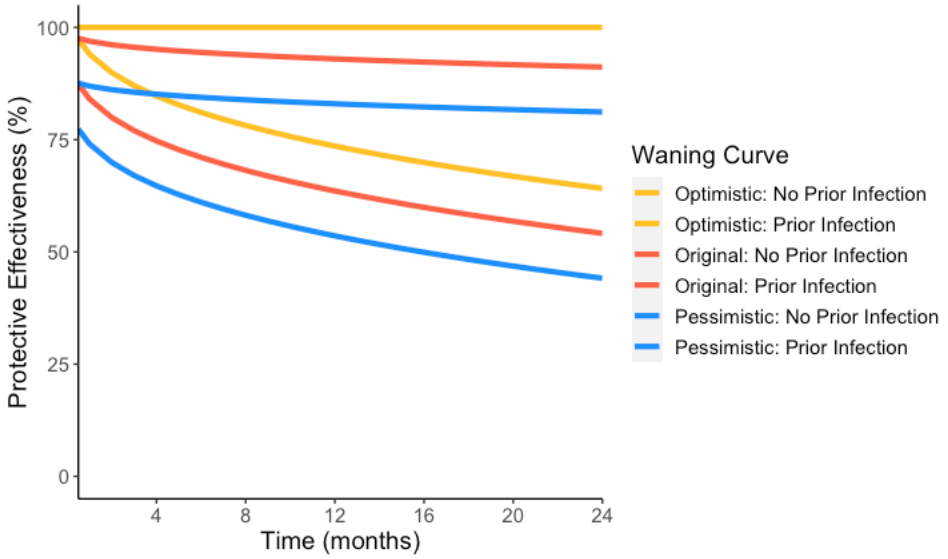

Figure S7: Protective effectiveness against severe COVID-19 generated from vaccine-induced and hybrid immunity with assumptions of pessimistic and optimistic vaccine effectiveness. After generating the absolute

predictions for vaccine-induced and hybrid immunity (red line), we adjusted the overall waning up (optimistic assumption; yellow line) or down (pessimistic assumption; blue line) by 10%.

High and low incidence of severe COVID-19

We conducted a sensitivity analysis on severe COVID-19 incidence, simulating higher or lower incidence scenarios. For this analysis, we applied multipliers of either 0.5x (lower incidence) and 2x (higher incidence) to risk group-specific estimates of severe COVID-19 incidence before running the model calibration. Then we simulated the three COVID-19 booster schedules in each risk-group cohort. The predicted annual risk of severe COVID-19 outcomes under each booster schedule are in the main manuscript (Figure 3). The predicted severe COVID-19 outcomes under each booster schedule are in Tables S17 and S18.

High and low seroprevalence

We conducted a sensitivity analysis on seroprevalence, simulating higher, lower, and 100% seroprevalence scenarios. For the higher and lower seroprevalence scenarios, we incorporated a 25% relative increase (higher seroprevalence) or decrease (lower seroprevalence) to the seroprevalence estimates in each risk group before running the model calibration. Then we calibrated the model and simulated the three COVID-19 booster schedules in each risk-group cohort. The predicted annual risk of severe COVID-19 outcomes under each booster schedule for the 100% seroprevalence sensitivity analysis is in the main manuscript (Figure 3) The predicted severe COVID-19 outcomes under each booster schedule are in Tables S19-S21.

Five-year time horizon

We conducted a sensitivity analysis simulating the impact of different booster vaccination schedules over a five-year time horizon instead of two years. We simulated the same three vaccination strategies from the main analysis: i) one-time booster at the start of the simulation (base case); ii) one-time booster followed by annual boosters (total of 5 doses); and iii) one-time booster followed by boosters every 6 months (total of 10 doses). We assumed that waning of protection remained fixed after 24 months. The predicted severe COVID-19 outcomes under each booster schedule are in Table S22.

Delayed vaccine administration

We conducted a sensitivity analysis simulating delayed vaccine administration, with the administration of boosters over a 6-month period rather than a 3-month period. We simulated the three COVID-19 booster schedules in each risk group cohort. The predicted severe COVID-19 outcomes under each booster schedule are in Table S23.

Higher sub-clinical infection

We conducted a sensitivity analysis with a higher proportion of sub-clinical COVID-19 infections. We applied a 2x multiplier to the risk group-specific non-severe infection multipliers. Then we simulated the three COVID-19 booster schedules in each risk-group cohort. The predicted severe COVID-19 outcomes under each booster schedule are in Table S24.

Lower vaccine effectiveness after first dose

We conducted a sensitivity analysis simulating lower vaccine effectiveness in subsequent booster doses after the first dose (applicable to annual and semiannual booster schedules only). Starting with the second booster dose, every vaccine dose that was administered had protection reset to

waned protection at month 3 instead of resetting waning completely at time point 0. Then we simulated the COVID-19 booster schedules in each risk group cohort. The full reporting of results for severe COVID-19 outcomes under each booster schedule are in Table S25.

Lower vaccine coverage
The primary model was formulated as a static model with perfect vaccine uptake; therefore, population-level benefit would scale with vaccine coverage.

Scenario analysis: Novel variants
We repeated the primary analysis under different scenarios for emergence of novel variants with immune evasion (summarized in Figure 1A). In scenario 1, a novel variant is introduced at the start of the simulation. In scenario 2, a novel variant is introduced at the start of Year 2 of the simulation. In scenario 3, a novel variant 1 is introduced at the start of the simulation, and a novel variant 2 is introduced at the start of Year 2. In scenario 4, the novel variant circulation is the same as outlined in scenario 3, but this time, the vaccines administered are targeted to the variant (two distinct vaccine formulations, akin to the seasonally targeted influenza vaccine) allowing for additional restoration of protection. Novel variants were introduced over a 3-month period. Variants are modeled under two different immune evasion scenarios: i) absolute protection from vaccine-induced or hybrid protection against severe COVID-19 is reduced by 10% with circulation of the novel variant, due to immune evasion; and ii) absolute protection is reduced by 10%, and rate of waning increases by 5% with circulation of the novel variant. In scenario 3 and 4, emergence of variant 2 led to an additional reduction in absolute protection in the population, beyond the initial reduction experienced during emergence of variant 1. In scenario 4, the variant-targeted vaccine restored the protection lost due to immune evasion of the new variant for vaccine-induced protection and partially restored protection for those with hybrid immunity. Infection with the currently circulating variant restored full hybrid immunity. A full description of modeling of variants and vaccine effects for scenario 3 and 4 (version 1) are found in Figures S8-S9. We simulated 8 total scenarios, with 4 variant scenarios and 2 immune evasion scenarios. We did not simulate variants with higher infectiousness or severity.

The predicted annual risk of severe COVID-19 outcomes under each booster schedule for each of the novel variant scenario analyses are in Figures S12-S13.

A. Original Variant Period (for reference)

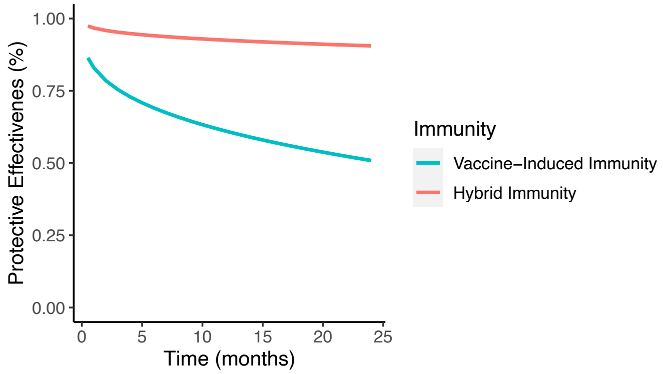

B. Novel Variant 1 Period

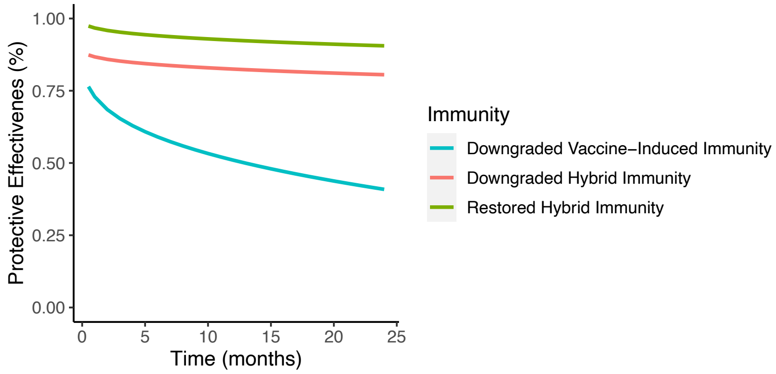

C. Novel Variant 2 Period

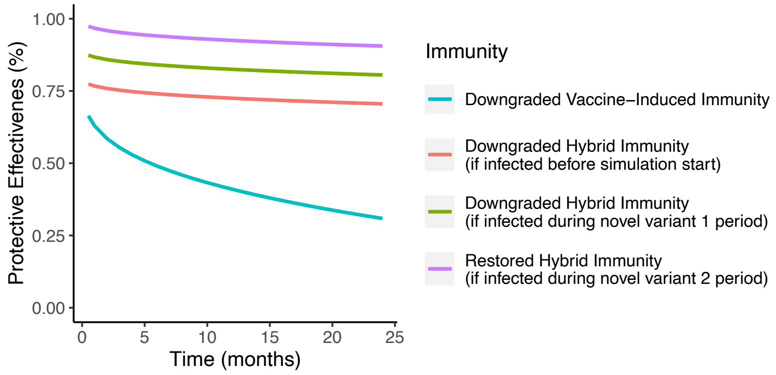

Figure S8: Novel variant scenario 3 (version 1) protective effectiveness against severe COVID-19. For scenario 3, we plot estimates for protective effectiveness during period of novel variant 1 (panel B) and novel variant 2 (panel C), including reference of the original variant (panel A).

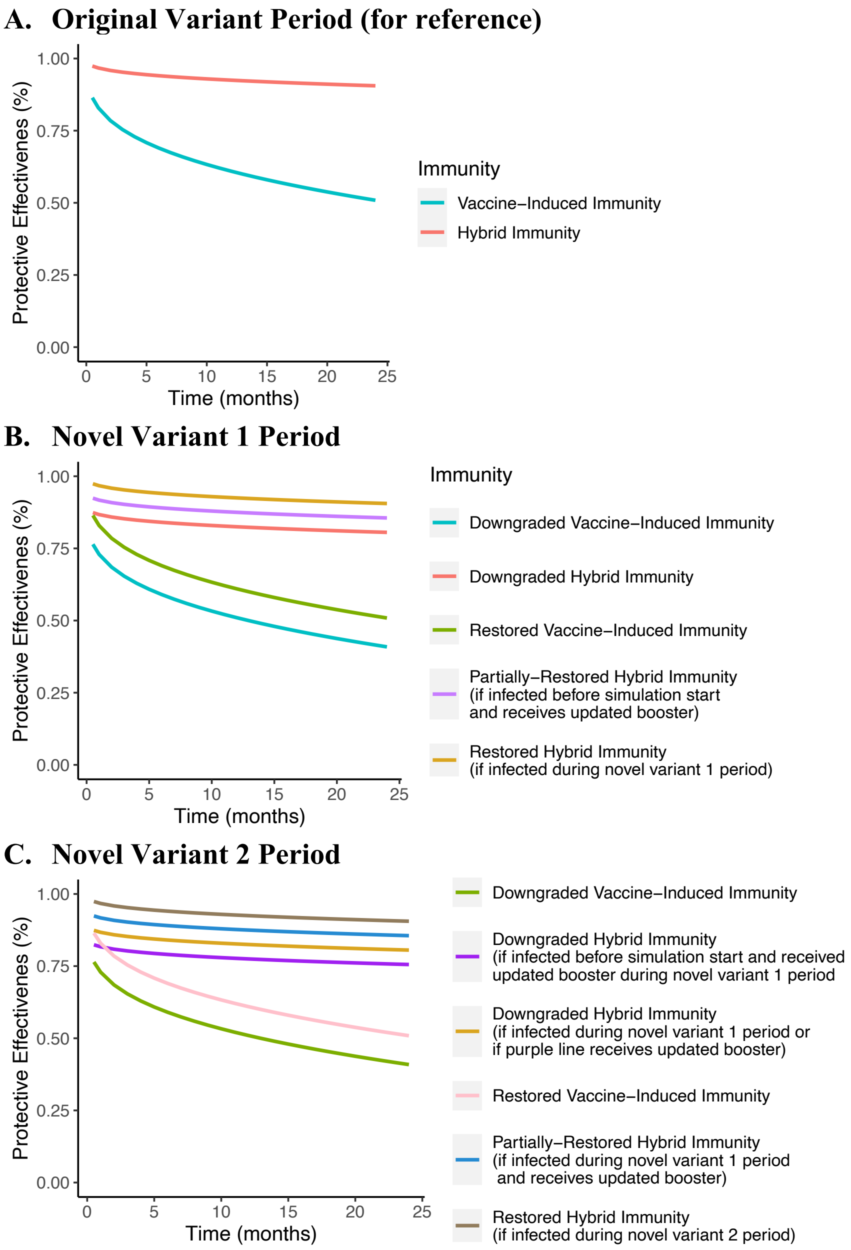

Figure S9: Novel variant scenario 4 (version 1) protective effectiveness against severe COVID-19. For scenario 4, we plot estimates for protective effectiveness during period of novel variant 1 (panel B) and novel variant 2 (panel C) with use of variant-targeted vaccine, including reference of the original variant (panel A).

<u>Scenario analysis: Dynamic transmission model</u>

In this scenario analysis, we repeated the primary analysis using a dynamic transmission model, which accounted for the indirect effects of vaccination on transmission. The objective was to determine to what extent booster vaccination strategies affected transmission and, by extension, risk of severe COVID-19, especially in high-risk groups. The dynamic model had key modifications from the primary microsimulation model, with the following governing equation.

$$Prob\_nonsevere\_infection(i,j,t) = \lambda_{all} * \beta_j * \left(1 - PE_{i,j,t}\right) * \sum_{k=1}^{5} C_{j,k} * \frac{I_{k,t-1}}{N_k}$$

$$\begin{aligned} &i = individual \\ &j = age\ group\ of\ individual\ i \\ &k = age\ group\ of\ others \\ &t = time \end{aligned}$$

The following is a summary of the differences in the dynamic transmission model compared to the primary model. First, the 'force of infection' term was formulated to be directly related to the number of SARS-CoV-2 infections in the population in the prior time step (week). This additional term of $\frac{I_{k,t-1}}{N_k}$ was applied to estimate the probability of SARS-CoV-2 infection (and severe COVID-19), where $I_{k,t-1}$ is the number of infections during the prior week in age group k and N_k is the population size in age group k. In a sensitivity analysis, we also tested a daily time step. We applied an age-based contact matrix to account for heterogeneous mixing by age group, using term $C_{j,k}$ to account for the number of contacts C between an individual of age group j with another age group k in the United States (see Table S6)[20]. These terms were summed across age groups to be the expected number of infectious contacts for an individual in age group j at time t. Second, a β_j term (transmission coefficient for age group j) was added to account for differences in transmission risk by age group, in order to support calibration of the model to observed age-specific COVID-19 incidence. Third, the simulated population included all age groups (addition of children, 0-17 years) and unvaccinated individuals. We used 100% minus the age-specific coverage estimates of primary series vaccine completion to estimate the proportion of unvaccinated persons[11]. We generated additional protective effectiveness and waning curves for children (0-17 years), unvaccinated individuals (with prior infection), and applied data on protection against clinical cases to protection against infection. Model inputs and assumptions for the 0–17-year age group can be found in Table S7. Fourth, we simulated a total population of 10 million, ensuring that age- and immunocompromised status reflects the United States population. Table S7 includes assumed demography, estimates for age-specific coverage of uptake of vaccine strategies, and risk of being immunocompromised[18,21]. Fifth, while the model was calibrated to match observed, age-specific non-severe COVID-19 outcomes at baseline (time 0), the model was not calibrated to match a defined number of severe COVID-19 cases over the 2-year simulation period (see Table S8 for model calibration). We assumed immunocompetent and immunocompromised groups had the same transmission coefficients, and used a higher non-severe case multiplier to better calibrate the model. Under the described approach to calibration, the dynamic model estimated a modestly higher number of severe COVID-19 cases compared to the static model over the simulation period for the single booster scenario. Since the goal of this model was not to predict the trends in COVID-19 outcomes over time, but rather compare the potential impact of indirect effects under different vaccine strategies by risk group, this approach to calibration was kept to minimize introduction of additional assumptions. Overall, the relative comparison between risk groups under different vaccine strategies was more important than the absolute estimates of severe COVID-19 risk to determine the potential impact of indirect effects. Sixth, vaccine strategies were applied with imperfect uptake coverage by age- and immune status to reflect current values[11,12] (see Table S7).

We compared booster vaccination strategies in the following groups to determine the impact of indirect effects of vaccination: i) 75+ years and moderately/severely immunocompromised (most restrictive); ii) 65+ years and mildly and moderately/severely immunocompromised; and iii) all groups 18+ years (most inclusive). We compared these booster vaccination strategies under two uptake scenarios: i) realistic uptake modeling current up-to-date coverage of boosters; and ii) optimistic uptake with higher coverage. In all the population targeting strategies (18+, 65+, and 75+ years) and with any of the booster interventions (one-time, annual, semiannual), the first booster is distributed to everyone 18 years and older under the two coverage scenarios from Table S7. Subsequent doses (if applicable) are distributed based on their respective population targeting strategies and respective coverage scenarios. If present, the largest indirect effects from vaccination are expected with more inclusive vaccine strategies and optimistic coverage. Study outcomes were computed in among persons assigned to the booster vaccination strategies (i.e., excluding unvaccinated persons, or those who did not receive additional vaccination); this was done to improve comparability to the primary model.

The predicted annual risk of severe COVID-19 outcomes under each booster schedule for the dynamic transmission model analyses are in Figures S14-S15.

Table S6. Age-based contact matrix for dynamic transmission model.

		Individual				
		0-17 years	18-49 years	50-64 years	65-74 years	75+ years
**Contacts** [a]	0-17 years	8.35	2.88	0.83	0.31	0.14
	18-49 years	5.55	9.98	3.22	0.48	0.23
	50-64 years	1.99	2.96	2.93	0.56	0.21
	65-74 years	0.61	0.73	0.79	1.17	0.31
	75+ years	0.31	0.27	0.26	0.24	0.40

[a] These are average contacts per day; we adjust these for a week time step when applicable. Contact matrix based on published study.[20]

Table S7. Demographic characteristics of population for dynamic model.

Age Group	Population Proportion [21]	Immuno-compromised Prevalence[18,a]	Prior Infection Prevalence	Proportion Unvaccinated [11]	Current up-to-date Vaccination (Realistic Booster Coverage)[11,12]	Optimistic Booster Coverage
0-17 years	0.221	0.0285	0.824	59%	0%	0%
18-49 years	0.420	0.058	0.824	21.3%	17%	30%
50-64 years	0.191	0.099	0.658	15.7%	28%	40%
65-74 years	0.075	0.139	0.468	11.5%	45%	70%
75+ years	0.094	0.139	0.468	11.5%	45%	70%
Immuno-compromised (All) [b]	--	--	--	--	60%	70%

[a] We assumed that severe immunocompromised individuals are 15% of the total immunocompromised population.
[b]The immunocompromised population represent a subset of each age group using the age-specific immunocompromised prevalence estimates. The immunocompromised population was assumed to have the same level of prior infection (seroprevalence) and unvaccinated status as their respective immunocompetent age groups. We assume a higher level of current up-to-date and optimistic vaccination coverage for immunocompromised groups.

Table S8. Model calibration for dynamic transmission model with risk estimates for non-severe COVID-19.

Age Group	Monthly Incidence, per 100,000 persons (Observed)	Monthly Incidence, per 100,000 persons (Model)
0-17 years	552	556
18-49 years	552	555
50-64 years	439	441
65-74 years	320	320
75+ years	374	375

Model reporting checklist
We completed the CHEERS checklist, which is a reporting standards checklist that be applied for simulation studies, as a supplemental file. We marked economic related items as N/A. We completed the Nature journal editorial checklists.

Computing
R packages used in this study include 'tidyverse', 'reshape2', 'lubridate', 'scales', 'lme4', 'data.table', 'foreach', 'doParallel', and 'here'.

Appendix References

1. Bobrovitz N, Ware H, Ma X, et al. Protective effectiveness of previous SARS-CoV-2 infection and hybrid immunity against the omicron variant and severe disease: a systematic review and meta-regression. *Lancet Infect Dis*. 2023;23(5):556-567. doi:10.1016/S1473-3099(22)00801-5

2. Carazo S, Skowronski DM, Brisson M, et al. Effectiveness of previous infection-induced and vaccine-induced protection against hospitalisation due to omicron BA subvariants in older adults: a test-negative, case-control study in Quebec, Canada. *Lancet Healthy Longev*. 2023;4(8):e409-e420. doi:10.1016/S2666-7568(23)00099-5

3. Ferdinands JM, Rao S, Dixon BE, et al. Waning of vaccine effectiveness against moderate and severe covid-19 among adults in the US from the VISION network: test negative, case-control study. *BMJ*. 2022;379:e072141. doi:10.1136/bmj-2022-072141

4. Lin DY, Xu Y, Gu Y, et al. Effectiveness of Bivalent Boosters against Severe Omicron Infection. *N Engl J Med*. 2023;388(8):764-766. doi:10.1056/NEJMc2215471

5. Andeweg SP, de Gier B, Eggink D, et al. Protection of COVID-19 vaccination and previous infection against Omicron BA.1, BA.2 and Delta SARS-CoV-2 infections. *Nat Commun*. 2022;13(1):4738. doi:10.1038/s41467-022-31838-8

6. Britton A, Embi PJ, Levy ME, et al. Effectiveness of COVID-19 mRNA Vaccines Against COVID-19-Associated Hospitalizations Among Immunocompromised Adults During SARS-CoV-2 Omicron Predominance - VISION Network, 10 States, December 2021-August 2022. *MMWR Morb Mortal Wkly Rep*. 2022;71(42):1335-1342. doi:10.15585/mmwr.mm7142a4

7. Lin DY, Xu Y, Gu Y, et al. Effectiveness of Bivalent Boosters against Severe Omicron Infection. *N Engl J Med*. 2023;388(8):764-766. doi:10.1056/NEJMc2215471

8. CDC. Nationwide COVID-19 Infection-Induced Antibody Seroprevalence (Commercial laboratories). Centers for Disease Control and Prevention. Published March 28, 2020. Accessed May 15, 2023. https://covid.cdc.gov/covid-data-tracker

9. CDC. Rates of COVID-19 Cases and Deaths by Vaccination Status. Centers for Disease Control and Prevention. Published March 28, 2020. Accessed May 15, 2023. https://covid.cdc.gov/covid-data-tracker

10. Bahremand T, Yao JA, Mill C, Piszczek J, Grant JM, Smolina K. COVID-19 hospitalisations in immunocompromised individuals in the Omicron era: a population-based observational study using surveillance data in British Columbia, Canada. *Lancet Reg Health – Am*. 2023;20. doi:10.1016/j.lana.2023.100461

11. CDPH. California Vaccine Progress Dashboard. Accessed January 25, 2023. https://covid19.ca.gov/vaccination-progress-data/

12. Lu P jun, Zhou T, Santibanez TA, et al. COVID-19 Bivalent Booster Vaccination Coverage and Intent to Receive Booster Vaccination Among Adolescents and Adults — United States, November–December 2022. *MMWR Morb Mortal Wkly Rep*. 2023;72(7):190-198. doi:10.15585/mmwr.mm7207a5

13. COVID-19 Time-Series Metrics by County and State - California Health and Human Services Open Data Portal. Accessed May 15, 2023. https://data.chhs.ca.gov/dataset/covid-19-time-series-metrics-by-county-and-state

14. CDC. COVID-NET Laboratory-confirmed COVID-19 hospitalizations. Centers for Disease Control and Prevention. Published March 28, 2020. Accessed May 15, 2023. https://covid.cdc.gov/covid-data-tracker

15. CDC. COVID-19 Vaccinations in the United States. Centers for Disease Control and Prevention. Published March 28, 2020. Accessed May 15, 2023. https://covid.cdc.gov/covid-data-tracker

16. Population of the U.S. by sex and age 2021. Statista. Accessed May 15, 2023. https://www.statista.com/statistics/241488/population-of-the-us-by-sex-and-age/

17. Risk for COVID-19 Infection, Hospitalization, and Death By Age Group. Centers for Disease Control and Prevention. Published February 11, 2020. Accessed May 15, 2023. https://www.cdc.gov/coronavirus/2019-ncov/covid-data/investigations-discovery/hospitalization-death-by-age.html

18. Patel M, Chen J, Kim S, et al. Analysis of MarketScan Data for Immunosuppressive Conditions and Hospitalizations for Acute Respiratory Illness, United States - Volume 26, Number 8—August 2020 - Emerging Infectious Diseases journal - CDC. doi:10.3201/eid2608.191493

19. Schumacher S, Presiado M, 2023. KFF COVID-19 Vaccine Monitor: January 2023. KFF. Published February 7, 2023. Accessed June 6, 2023. https://www.kff.org/coronavirus-covid-19/poll-finding/kff-covid-19-vaccine-monitor-january-2023/

20. Prem K, Cook AR, Jit M. Projecting social contact matrices in 152 countries using contact surveys and demographic data. *PLOS Comput Biol*. 2017;13(9):e1005697. doi:10.1371/journal.pcbi.1005697

21. Bureau UC. Exploring Age Groups in the 2020 Census. Census.gov. Accessed October 29, 2023. https://www.census.gov/library/visualizations/interactive/exploring-age-groups-in-the-2020-census.html

Supplemental Tables and Figures

Table S9: Number of non-severe COVID-19 cases, risk, and number needed to treat to avert non-severe COVID-19 in six risk groups with different frequencies of COVID-19 booster vaccination.
Table S10: Number of severe COVID-19 cases, risk, and number needed to treat to avert severe COVID-19 in four age groups among the mild immunocompromised population with different frequencies of COVID-19 booster vaccination.
Table S11: Number of severe COVID-19 cases, risk, and number needed to treat to avert severe COVID-19 in four age groups among the moderate/severe immunocompromised population with different frequencies of COVID-19 booster vaccination.
Table S12: Number of severe COVID-19 cases and risk in six risk groups with no additional COVID-19 booster vaccination.
Table S13: Sensitivity analysis of pessimistic waning on the number of severe COVID-19 cases, risk, and number needed to treat to avert severe COVID-19 in six risk groups with different frequencies of COVID-19 booster vaccination.
Table S14: Sensitivity analysis of optimistic waning on the number of severe COVID-19 cases, risk, and number needed to treat to avert severe COVID-19 in six risk groups with different frequencies of COVID-19 booster vaccination.
Table S15: Sensitivity analysis of pessimistic vaccine effectiveness on the number of severe COVID-19 cases, risk, and number needed to treat to avert severe COVID-19 in six risk groups with different frequencies of COVID-19 booster vaccination.
Table S16: Sensitivity analysis of optimistic vaccine effectiveness on the number of severe COVID-19 cases, risk, and number needed to treat to avert severe COVID-19 in six risk groups with different frequencies of COVID-19 booster vaccination.
Table S17: Sensitivity analysis of higher severe COVID-19 incidence on the number of severe COVID-19 cases, risk, and number needed to treat to avert severe COVID-19 in six risk groups with different frequencies of COVID-19 booster vaccination.
Table S18: Sensitivity analysis of lower severe COVID-19 incidence on the number of severe COVID-19 cases, risk, and number needed to treat to avert severe COVID-19 in six risk groups with different frequencies of COVID-19 booster vaccination.
Table S19: Sensitivity analysis of lower seroprevalence on the number of severe COVID-19 cases, risk, and number needed to treat to avert severe COVID-19 in six risk groups with different frequencies of COVID-19 booster vaccination.
Table S20: Sensitivity analysis of higher seroprevalence on the number of severe COVID-19 cases, risk, and number needed to treat to avert severe COVID-19 in six risk groups with different frequencies of COVID-19 booster vaccination.
Table S21: Sensitivity analysis of 100% seroprevalence on the number of severe COVID-19 cases, risk, and number needed to treat to avert severe COVID-19 in six risk groups with different frequencies of COVID-19 booster vaccination.
Table S22: Sensitivity analysis of a five-year simulation period on the number of severe COVID-19 cases, risk, and number needed to treat to avert severe COVID-19 in six risk groups with different frequencies of COVID-19 booster vaccination.
Table S23: Sensitivity analysis of delayed vaccination administration on the number of severe COVID-19 cases, risk, and number needed to treat to avert severe COVID-19 in six risk groups with different frequencies of COVID-19 booster vaccination.

Table S24: Sensitivity analysis of higher sub-clinical infection on the number of severe COVID-19 cases, risk, and number needed to treat to avert severe COVID-19 in six risk groups with different frequencies of COVID-19 booster vaccination.
Table S25: Sensitivity analysis of lower vaccine effectiveness for subsequent doses after the first dose on the number of severe COVID-19 cases, risk, and number needed to treat to avert severe COVID-19 in six risk groups with different frequencies of COVID-19 booster vaccination.

Figure S10: Monthly incidence of severe COVID-19 in four age groups and two immunocompromised groups over a two-year simulation period with different frequencies of COVID-19 booster vaccination.
Figure S11: Risk of severe COVID-19 over time by baseline risk and waning protection.
Figure S12: Scenario analysis on emergence of novel SARS-CoV-2 variants with immune evasion (10% reduction in immunity) comparing severe COVID-19 risk with different frequencies of COVID-19 booster vaccination.
Figure S13: Scenario analysis on emergence of novel SARS-CoV-2 variants with immune evasion (10% absolute reduction and 5% increased rate of waning) comparing severe COVID-19 risk with different frequencies of COVID-19 booster vaccination.
Figure S14: Scenario analysis using a dynamic transmission model under realistic coverage assumptions to estimate the impact of indirect effects on COVID-19 booster vaccination strategies in four age groups and two immunocompromised groups.
Figure S15: Scenario analysis using a dynamic transmission model under optimistic coverage assumptions to estimate the impact of indirect effects on COVID-19 booster vaccination strategies in four age groups and two immunocompromised groups.

Table S9: Number of non-severe COVID-19 cases, risk, and number needed to treat to avert non-severe COVID-19 in six risk groups with different frequencies of COVID-19 booster vaccination.

	Total non-severe COVID-19 cases[a]	Absolute annual risk of non-severe COVID-19	Annual risk reduction of non-severe COVID-19		NNT to avert non-severe COVID-19 case[a]
		(cases per 100,000; UI)	Absolute risk averted (cases per 100,000)	Relative risk averted (%)	
One-time booster[b]					
18-49 years	439,365	21,968 (15,896-29,893)	--	--	--
50-64 years	355,734	17,787 (12,572-25,166)	--	--	--
65-74 years	265,239	13,262 (9,139-17,272)	--	--	--
75+ years	303,767	15,188 (10,530-19,719)	--	--	--
Immunocompromised (Mild)	358,657	17,933 (12,797-24,261)	--	--	--
Immunocompromised (Moderate/Severe)	363,323	18,166 (12,858-24,605)	--	--	--
Annual booster					
18-49 years	367,029	18,351 (13,220-24,656)	3,617	16%	14
50-64 years	300,898	15,045 (10,768-20,798)	2,742	15%	19
65-74 years	217,293	10,865 (7,906-14,030)	2,397	18%	21
75+ years	250,000	12,500 (9,116-16,127)	2,688	18%	19
Immunocompromised (Mild)	313,786	15,689 (11,257-20,898)	2,244	13%	23
Immunocompromised (Moderate/Severe)	313,923	15,696 (11,274-20,822)	2,470	14%	21
Semiannual booster (every 6 months)					
18-49 years	301,214	15,061 (10,764-20,129)	6,908	31%	8
50-64 years	246,275	12,314 (8,849-17,085)	5,473	31%	10
65-74 years	176,486	8,824 (6,426-11,413)	4,438	33%	12
75+ years	203,741	10,187 (7,443-13,170)	5,001	33%	10
Immunocompromised (Mild)	270,767	13,538 (9,684-18,073)	4,395	25%	12
Immunocompromised (Moderate/Severe)	268,496	13,425 (9,569-17,736)	4,741	26%	11

[a]Estimated over 2-year simulation period in population of 1 million persons.
[b]One-time booster is the baseline intervention for risk reduction calculations.

Table S10: Number of severe COVID-19 cases, risk, and number needed to treat to avert severe COVID-19 in four age groups among the mild immunocompromised population with different frequencies of COVID-19 booster vaccination.

	Total severe COVID-19 cases[a]	Absolute annual risk of severe COVID-19	Annual risk reduction of severe COVID-19		% Averted severe COVID-19		NNT to avert severe COVID-19 case[a]
		(cases per 100,000; 95% UI)	Absolute risk averted (cases per 100,000)	Relative risk averted (%)	No Prior Infection	Prior Infection	
One-time booster[b]							
18-49 years	5,851	293 (267-339)	--	--	--	--	--
50-64 years	11,746	587 (539-668)	--	--	--	--	--
65-74 years	30,065	1,503 (1,411-1,612)	--	--	--	--	--
75+ years	80,148	4,007 (3,763-4,296)	--	--	--	--	--
Annual booster							
18-49 years	5,441	272 (243-322)	21	7%	48%	52%	2,440
50-64 years	10,863	543 (490-627)	44	8%	73%	27%	1,133
65-74 years	27,311	1,366 (1,270-1,492)	138	9%	83%	17%	364
75+ years	73,147	3,657 (3,391-4,033)	350	9%	82%	18%	143
Semiannual booster (every 6 months)							
18-49 years	5,173	258 (224-309)	34	12%	49%	51%	1,475
50-64 years	10,070	504 (445-602)	84	14%	71%	29%	597
65-74 years	25,180	1,259 (1,148-1,421)	244	16%	83%	17%	205
75+ years	67,745	3,387 (3,076-3,841)	620	15%	82%	18%	81

[a]Estimated over 2-year simulation period in population of 1 million persons.
[b]One-time booster is the baseline intervention for risk reduction calculations.

Table S11: Number of severe COVID-19 cases, risk, and number needed to treat to avert severe COVID-19 in four age groups among the moderate/severe immunocompromised population with different frequencies of COVID-19 booster vaccination.

	Total severe COVID-19 cases[a]	Absolute annual risk of severe COVID-19	Annual risk reduction of severe COVID-19		% Averted severe COVID-19		NNT to avert severe COVID-19 case[a]
		(cases per 100,000; 95% UI)	Absolute risk averted (cases per 100,000)	Relative risk averted (%)	No Prior Infection	Prior Infection	
One-time booster[b]							
18-49 years	6,153	308 (278-353)	--	--	--	--	--
50-64 years	12,498	625 (570-715)	--	--	--	--	--
65-74 years	31,891	1,595 (1,488-1,724)	--	--	--	--	--
75+ years	84,909	4,245 (3,947-4,624)	--	--	--	--	--
Annual booster							
18-49 years	5,353	268 (243-308)	40	13%	31%	69%	1,250
50-64 years	10,829	541 (491-622)	83	13%	51%	49%	600
65-74 years	27,471	1,374 (1,276-1,489)	221	14%	69%	31%	227
75+ years	73,542	3,677 (3,402-4,024)	568	13%	68%	32%	88
Semiannual booster (every 6 months)							
18-49 years	4,842	242 (215-280)	66	21%	32%	68%	763
50-64 years	9,649	482 (435-565)	142	23%	52%	48%	352
65-74 years	24,382	1,219 (1,124-1,345)	375	24%	71%	29%	134
75+ years	65,716	3,286 (3,017-3,642)	960	23%	70%	30%	53

[a]Estimated over 2-year simulation period in population of 1 million persons.
[b]One-time booster is the baseline intervention for risk reduction calculations.

Table S12: Number of severe COVID-19 cases and risk in six risk groups with no additional COVID-19 booster vaccination.

	Total severe COVID-19 cases[a]	Absolute annual risk of severe COVID-19 (cases per 100,000; UI)
No booster given during simulation[b]		
18-49 years	2,249	113 (99-144)
50-64 years	4,620	231 (209-273)
65-74 years	12,062	603 (569-651)
75+ years	31,967	1,598 (1,504-1,730)
Immunocompromised (Mild)	28,094	1,405 (1,312-1,504)
Immunocompromised (Moderate/Severe)	31,276	1,564 (1,461-1,704)

[a]Estimated over 2-year simulation period in population of 1 million persons.
[b]No boosters given during simulation, although persons have received at least one monovalent booster dose prior to the initiation of the simulation.

Table S13: Sensitivity analysis of pessimistic waning on the number of severe COVID-19 cases, risk, and number needed to treat to avert severe COVID-19 in six risk groups with different frequencies of COVID-19 booster vaccination.

	Total severe COVID-19 cases[a]	Absolute annual risk of severe COVID-19 (cases per 100,000; UI)	Annual risk reduction of severe COVID-19		NNT to avert severe COVID-19 case[a]
			Absolute risk averted (cases per 100,000)	Relative risk averted (%)	
One-time booster[b]					
18-49 years	2,124	106 (91-140)	--	--	--
50-64 years	4,433	222 (197-284)	--	--	--
65-74 years	11,423	571 (528-656)	--	--	--
75+ years	30,451	1,523 (1,409-1,764)	--	--	--
Immunocompromised (Mild)	27,086	1,354 (1,256-1,489)	--	--	--
Immunocompromised (Moderate/Severe)	28,422	1,421 (1,300-1,577)	--	--	--
Annual booster					
18-49 years	1,692	85 (72-104)	22	20%	2,315
50-64 years	3,504	175 (158-210)	46	21%	1,077
65-74 years	9,057	453 (429-491)	118	21%	423
75+ years	24,390	1,220 (1,150-1,328)	303	20%	165
Immunocompromised (Mild)	23,667	1,183 (1,089-1,311)	171	13%	293
Immunocompromised (Moderate/Severe)	23,711	1,186 (1,091-1,307)	236	17%	213
Semiannual booster (every 6 months)					
18-49 years	1,351	68 (58-81)	39	36%	1,294
50-64 years	2,871	144 (132-164)	78	35%	641
65-74 years	7,463	373 (349-395)	198	35%	253
75+ years	20,104	1,005 (951-1,061)	517	34%	97
Immunocompromised (Mild)	21,261	1,063 (968-1,197)	291	22%	172
Immunocompromised (Moderate/Severe)	20,656	1,033 (948-1,140)	388	27%	129

[a]Estimated over 2-year simulation period in population of 1 million persons.
[b]One-time booster is the baseline intervention for risk reduction calculations.

Table S14: Sensitivity analysis of optimistic waning on the number of severe COVID-19 cases, risk, and number needed to treat to avert severe COVID-19 in six risk groups with different frequencies of COVID-19 booster vaccination.

	Total severe COVID-19 cases[a]	Absolute annual risk of severe COVID-19 (cases per 100,000; UI)	Annual risk reduction of severe COVID-19: Absolute risk averted (cases per 100,000)	Annual risk reduction of severe COVID-19: Relative risk averted (%)	NNT to avert severe COVID-19 case[a]
One-time booster[b]					
18-49 years	1,776	88 (74-120)	--	--	--
50-64 years	3,620	181 (162-212)	--	--	--
65-74 years	9,688	484 (436-527)	--	--	--
75+ years	25,779	1,289 (1,155-1,402)	--	--	--
Immunocompromised (Mild)	24,724	1,236 (1,149-1,351)	--	--	--
Immunocompromised (Moderate/Severe)	26,058	1,303 (1,217-1,420)	--	--	--
Annual booster					
18-49 years	1,657	83 (71-120)	6	7%	8,404
50-64 years	3,305	165 (153-201)	16	9%	3,175
65-74 years	8,734	437 (411-472)	48	10%	1,049
75+ years	23,393	1,170 (1,102-1,255)	119	9%	420
Immunocompromised (Mild)	23,642	1,182 (1,084-1,330)	54	4%	925
Immunocompromised (Moderate/Severe)	23,612	1,181 (1,088-1,318)	122	9%	409
Semiannual booster (every 6 months)					
18-49 years	1,544	78 (65-119)	12	13%	4,311
50-64 years	3,023	151 (140-188)	30	16%	1,676
65-74 years	7,853	393 (372-428)	92	19%	545
75+ years	21,114	1,056 (1,005-1,142)	233	18%	215
Immunocompromised (Mild)	22,571	1,129 (1,010-1,311)	108	9%	465
Immunocompromised (Moderate/Severe)	21,748	1,087 (985-1,245)	216	17%	233

[a]Estimated over 2-year simulation period in population of 1 million persons.
[b]One-time booster is the baseline intervention for risk reduction calculations.

Table S15: Sensitivity analysis of pessimistic vaccine effectiveness on the number of severe COVID-19 cases, risk, and number needed to treat to avert severe COVID-19 in six risk groups with different frequencies of COVID-19 booster vaccination.

	Total severe COVID-19 cases[a]	Absolute annual risk of severe COVID-19 (cases per 100,000; UI)	Annual risk reduction of severe COVID-19		NNT to avert severe COVID-19 case[a]
			Absolute risk averted (cases per 100,000)	Relative risk averted (%)	
One-time booster[b]					
18-49 years	2,081	104 (90-124)	--	--	--
50-64 years	4,179	209 (189-243)	--	--	--
65-74 years	10,752	538 (503-583)	--	--	--
75+ years	29,029	1,451 (1,356-1,562)	--	--	--
Immunocompromised (Mild)	26,226	1,311 (1,217-1,413)	--	--	--
Immunocompromised (Moderate/Severe)	27,384	1,369 (1,268-1,482)	--	--	--
Annual booster					
18-49 years	1,929	96 (83-116)	8	7%	6,579
50-64 years	3,792	190 (170-226)	19	9%	2,584
65-74 years	9,672	484 (448-533)	54	10%	926
75+ years	26,199	1,310 (1,216-1,443)	142	10%	354
Immunocompromised (Mild)	24,530	1,227 (1,122-1,352)	85	6%	590
Immunocompromised (Moderate/Severe)	24,464	1,223 (1,124-1,342)	146	11%	343
Semiannual booster (every 6 months)					
18-49 years	1,798	90 (76-112)	14	14%	3,534
50-64 years	3,494	175 (153-214)	34	16%	1,460
65-74 years	8,832	442 (402-500)	96	18%	521
75+ years	23,952	1,198 (1,092-1,360)	254	17%	197
Immunocompromised (Mild)	23,164	1,158 (1,039-1,311)	153	12%	327
Immunocompromised (Moderate/Severe)	22,471	1,124 (1,016-1,254)	246	18%	204

[a]Estimated over 2-year simulation period in population of 1 million persons.
[b]One-time booster is the baseline intervention for risk reduction calculations.

Table S16: Sensitivity analysis of optimistic vaccine effectiveness on the number of severe COVID-19 cases, risk, and number needed to treat to avert severe COVID-19 in six risk groups with different frequencies of COVID-19 booster vaccination.

	Total severe COVID-19 cases[a]	Absolute annual risk of severe COVID-19 (cases per 100,000; UI)	Annual risk reduction of severe COVID-19		NNT to avert severe COVID-19 case[a]
			Absolute risk averted (cases per 100,000)	Relative risk averted (%)	
One-time booster[b]					
18-49 years	1,500	75 (0-372)	--	--	--
50-64 years	3,567	178 (138-238)	--	--	--
65-74 years	10,049	502 (427-593)	--	--	--
75+ years	26,374	1,319 (1,092-1,574)	--	--	--
Immunocompromised (Mild)	25,023	1,251 (1,176-1,377)	--	--	--
Immunocompromised (Moderate/Severe)	27,299	1,365 (1,257-1,565)	--	--	--
Annual booster					
18-49 years	1,233	62 (0-118)	13	18%	3,746
50-64 years	2,858	143 (109-164)	35	20%	1,411
65-74 years	7,931	397 (328-442)	106	21%	473
75+ years	21,056	1,053 (857-1,171)	266	20%	189
Immunocompromised (Mild)	21,896	1,095 (1,028-1,222)	156	12%	320
Immunocompromised (Moderate/Severe)	22,228	1,111 (1,036-1,233)	254	19%	198
Semiannual booster (every 6 months)					
18-49 years	991	50 (0-58)	25	34%	1,965
50-64 years	2,294	115 (81-131)	64	36%	786
65-74 years	6,181	309 (225-346)	193	38%	259
75+ years	16,462	823 (599-925)	496	38%	101
Immunocompromised (Mild)	19,388	969 (900-1,105)	282	23%	178
Immunocompromised (Moderate/Severe)	18,702	935 (879-1,019)	430	31%	117

[a]Estimated over 2-year simulation period in population of 1 million persons.
[b]One-time booster is the baseline intervention for risk reduction calculations.

Table S17: Sensitivity analysis of higher severe COVID-19 incidence on the number of severe COVID-19 cases, risk, and number needed to treat to avert severe COVID-19 in six risk groups with different frequencies of COVID-19 booster vaccination.

	Total severe COVID-19 cases[a]	Absolute annual risk of severe COVID-19 (cases per 100,000; UI)	Annual risk reduction of severe COVID-19		NNT to avert severe COVID-19 case[a]
			Absolute risk averted (cases per 100,000)	Relative risk averted (%)	
One-time booster[b]					
18-49 years	3,331	167 (140-236)	--	--	--
50-64 years	6,878	344 (308-434)	--	--	--
65-74 years	18,438	922 (860-1,025)	--	--	--
75+ years	48,108	2,405 (2,234-2,660)	--	--	--
Immunocompromised (Mild)	46,441	2,322 (2,124-2,612)	--	--	--
Immunocompromised (Moderate/Severe)	49,311	2,466 (2,223-2,800)	--	--	--
Annual booster					
18-49 years	2,973	149 (126-204)	18	11%	2,794
50-64 years	6,044	302 (273-376)	42	12%	1,200
65-74 years	16,134	807 (753-879)	115	12%	435
75+ years	42,292	2,115 (1,986-2,308)	291	13%	172
Immunocompromised (Mild)	43,384	2,169 (1,948-2,497)	153	7%	328
Immunocompromised (Moderate/Severe)	43,681	2,184 (1,962-2,485)	282	11%	178
Semiannual booster (every 6 months)					
18-49 years	2,591	130 (112-177)	37	22%	1,352
50-64 years	5,302	265 (241-325)	79	23%	635
65-74 years	14,058	703 (661-759)	219	24%	229
75+ years	37,158	1,858 (1,753-2,008)	548	23%	92
Immunocompromised (Mild)	40,764	2,038 (1,799-2,407)	284	12%	177
Immunocompromised (Moderate/Severe)	39,606	1,980 (1,765-2,270)	485	20%	104

[a]Estimated over 2-year simulation period in population of 1 million persons.
[b]One-time booster is the baseline intervention for risk reduction calculations.

Table S18: Sensitivity analysis of lower severe COVID-19 incidence on the number of severe COVID-19 cases, risk, and number needed to treat to avert severe COVID-19 in six risk groups with different frequencies of COVID-19 booster vaccination.

	Total severe COVID-19 cases[a]	Absolute annual risk of severe COVID-19 (cases per 100,000; UI)	Annual risk reduction of severe COVID-19		NNT to avert severe COVID-19 case[a]
			Absolute risk averted (cases per 100,000)	Relative risk averted (%)	
One-time booster[b]					
18-49 years	1,052	53 (46-68)	--	--	--
50-64 years	2,198	110 (100-129)	--	--	--
65-74 years	5,598	280 (266-300)	--	--	--
75+ years	15,216	761 (727-805)	--	--	--
Immunocompromised (Mild)	13,697	685 (640-736)	--	--	--
Immunocompromised (Moderate/Severe)	14,495	725 (678-786)	--	--	--
Annual booster					
18-49 years	883	44 (39-56)	8	16%	5,918
50-64 years	1,823	91 (83-108)	19	17%	2,667
65-74 years	4,693	235 (223-251)	45	16%	1,105
75+ years	12,771	639 (610-676)	122	16%	409
Immunocompromised (Mild)	12,392	620 (575-683)	65	10%	767
Immunocompromised (Moderate/Severe)	12,369	618 (574-676)	106	15%	471
Semiannual booster (every 6 months)					
18-49 years	747	37 (33-48)	15	29%	3,279
50-64 years	1,551	78 (70-90)	32	29%	1,546
65-74 years	3,998	200 (188-213)	80	29%	625
75+ years	10,833	542 (519-574)	219	29%	229
Immunocompromised (Mild)	11,380	569 (518-646)	116	17%	432
Immunocompromised (Moderate/Severe)	10,983	549 (504-609)	176	24%	285

[a]Estimated over 2-year simulation period in population of 1 million persons.
[b]One-time booster is the baseline intervention for risk reduction calculations.

Table S19: Sensitivity analysis of lower seroprevalence on the number of severe COVID-19 cases, risk, and number needed to treat to avert severe COVID-19 in six risk groups with different frequencies of COVID-19 booster vaccination.

	Total severe COVID-19 cases[a]	Absolute annual risk of severe COVID-19 (cases per 100,000; UI)	Annual risk reduction of severe COVID-19		NNT to avert severe COVID-19 case[a]
			Absolute risk averted (cases per 100,000)	Relative risk averted (%)	
One-time booster[b]					
18-49 years	1,794	90 (82-101)	--	--	--
50-64 years	3,968	198 (181-217)	--	--	--
65-74 years	10,374	519 (490-547)	--	--	--
75+ years	27,692	1,385 (1,321-1,464)	--	--	--
Immunocompromised (Mild)	24,926	1,246 (1,188-1,315)	--	--	--
Immunocompromised (Moderate/Severe)	26,305	1,315 (1,248-1,388)	--	--	--
Annual booster					
18-49 years	1,560	78 (70-86)	12	13%	4,274
50-64 years	3,371	169 (156-183)	30	15%	1,676
65-74 years	8,774	439 (417-462)	80	15%	625
75+ years	23,603	1,180 (1,133-1,232)	204	15%	245
Immunocompromised (Mild)	22,637	1,132 (1,070-1,210)	114	9%	437
Immunocompromised (Moderate/Severe)	22,662	1,133 (1,072-1,202)	182	14%	275
Semiannual booster (every 6 months)					
18-49 years	1,336	67 (61-74)	23	26%	2,184
50-64 years	2,903	145 (134-157)	53	27%	939
65-74 years	7,494	375 (359-396)	144	28%	348
75+ years	20,242	1,012 (973-1,054)	373	27%	135
Immunocompromised (Mild)	20,803	1,040 (967-1,140)	206	17%	243
Immunocompromised (Moderate/Severe)	20,151	1,008 (947-1,082)	308	23%	163

[a]Estimated over 2-year simulation period in population of 1 million persons.
[b]One-time booster is the baseline intervention for risk reduction calculations.

Table S20: Sensitivity analysis of higher seroprevalence on the number of severe COVID-19 cases, risk, and number needed to treat to avert severe COVID-19 in six risk groups with different frequencies of COVID-19 booster vaccination.

	Total severe COVID-19 cases[a]	Absolute annual risk of severe COVID-19 (cases per 100,000; UI)	Annual risk reduction of severe COVID-19		NNT to avert severe COVID-19 case[a]
			Absolute risk averted (cases per 100,000)	Relative risk averted (%)	
One-time booster[b]					
18-49 years	2,391	120 (97-128)	--	--	--
50-64 years	4,254	213 (189-271)	--	--	--
65-74 years	10,449	522 (497-616)	--	--	--
75+ years	27,791	1,390 (1,331-1,646)	--	--	--
Immunocompromised (Mild)	25,970	1,299 (1,235-1,491)	--	--	--
Immunocompromised (Moderate/Severe)	27,635	1,382 (1,306-1,600)	--	--	--
Annual booster					
18-49 years	2,032	102 (83-109)	18	15%	2,786
50-64 years	3,644	182 (163-230)	31	14%	1,640
65-74 years	8,874	444 (425-520)	79	15%	635
75+ years	23,783	1,189 (1,143-1,398)	200	14%	250
Immunocompromised (Mild)	24,019	1,201 (1,125-1,411)	98	8%	513
Immunocompromised (Moderate/Severe)	23,966	1,198 (1,130-1,396)	183	13%	273
Semiannual booster (every 6 months)					
18-49 years	1,745	87 (72-93)	32	27%	1,548
50-64 years	3,115	156 (141-194)	57	27%	878
65-74 years	7,599	380 (363-443)	143	27%	351
75+ years	20,494	1,025 (989-1,190)	365	26%	138
Immunocompromised (Mild)	22,416	1,121 (1,034-1,358)	178	14%	282
Immunocompromised (Moderate/Severe)	21,516	1,076 (1,005-1,268)	306	22%	164

[a]Estimated over 2-year simulation period in population of 1 million persons.
[b]One-time booster is the baseline intervention for risk reduction calculations.

Table S21: Sensitivity analysis of 100% seroprevalence on the number of severe COVID-19 cases, risk, and number needed to treat to avert severe COVID-19 in six risk groups with different frequencies of COVID-19 booster vaccination.

	Total severe COVID-19 cases[a]	Absolute annual risk of severe COVID-19 (cases per 100,000; UI)	Annual risk reduction of severe COVID-19		NNT to avert severe COVID-19 case[a]
			Absolute risk averted (cases per 100,000)	Relative risk averted (%)	
One-time booster[b]					
18-49 years	2,403	120 (117-128)	--	--	--
50-64 years	5,203	260 (255-272)	--	--	--
65-74 years	13,558	678 (671-700)	--	--	--
75+ years	36,870	1,843 (1,839-1,893)	--	--	--
Immunocompromised (Mild)	29,996	1,500 (1,449-1,551)	--	--	--
Immunocompromised (Moderate/Severe)	32,739	1,637 (1,621-1,655)	--	--	--
Annual booster					
18-49 years	2,045	102 (99-109)	18	15%	2,794
50-64 years	4,433	222 (216-231)	39	15%	1,299
65-74 years	11,431	572 (562-592)	106	16%	471
75+ years	31,331	1,567 (1,553-1,594)	277	15%	181
Immunocompromised (Mild)	28,564	1,428 (1,410-1,454)	72	5%	699
Immunocompromised (Moderate/Severe)	28,432	1,422 (1,405-1,439)	215	13%	233
Semiannual booster (every 6 months)					
18-49 years	1,747	87 (82-92)	33	27%	1,525
50-64 years	3,718	186 (180-195)	74	29%	674
65-74 years	9,709	485 (473-496)	192	28%	260
75+ years	26,458	1,323 (1,303-1,346)	521	28%	97
Immunocompromised (Mild)	27,394	1,370 (1,334-1,375)	130	9%	385
Immunocompromised (Moderate/Severe)	25,521	1,276 (1,253-1,307)	361	22%	139

[a]Estimated over 2-year simulation period in population of 1 million persons.
[b]One-time booster is the baseline intervention for risk reduction calculations.

Table S22: Sensitivity analysis of a five-year simulation period on the number of severe COVID-19 cases, risk, and number needed to treat to avert severe COVID-19 in six risk groups with different frequencies of COVID-19 booster vaccination.

	Total severe COVID-19 cases[a]	Absolute annual risk of severe COVID-19 (cases per 100,000; UI)	Annual risk reduction of severe COVID-19		NNT to avert severe COVID-19 case[a]
			Absolute risk averted (cases per 100,000)	Relative risk averted (%)	
One-time booster[b]					
18-49 years	4,693	94 (76-141)	--	--	--
50-64 years	9,488	190 (161-251)	--	--	--
65-74 years	24,828	497 (444-571)	--	--	--
75+ years	63,790	1,276 (1,142-1,473)	--	--	--
Immunocompromised (Mild)	61,048	1,221 (1,117-1,374)	--	--	--
Immunocompromised (Moderate/Severe)	70,131	1,403 (1,253-1,622)	--	--	--
Annual booster					
18-49 years	3,588	72 (59-106)	22	24%	905
50-64 years	7,219	144 (127-187)	45	24%	441
65-74 years	18,989	380 (352-424)	117	24%	172
75+ years	49,785	996 (927-1,109)	280	22%	72
Immunocompromised (Mild)	52,973	1,059 (941-1,242)	162	13%	124
Immunocompromised (Moderate/Severe)	53,646	1,073 (953-1,242)	330	24%	61
Semiannual booster (every 6 months)					
18-49 years	3,107	62 (51-89)	32	34%	631
50-64 years	6,280	126 (112-159)	64	34%	312
65-74 years	16,516	330 (307-364)	166	33%	121
75+ years	43,512	870 (816-956)	406	32%	50
Immunocompromised (Mild)	49,610	992 (864-1,191)	229	19%	88
Immunocompromised (Moderate/Severe)	48,090	962 (848-1,121)	441	31%	46

[a]Estimated over 5-year simulation period in population of 1 million persons.
[b]One-time booster is the baseline intervention for risk reduction calculations.

Table S23: Sensitivity analysis of delayed vaccine administration on the number of severe COVID-19 cases, risk, and number needed to treat to avert severe COVID-19 in six risk groups with different frequencies of COVID-19 booster vaccination.

	Total severe COVID-19 cases[a]	Absolute annual risk of severe COVID-19 (cases per 100,000; UI)	Annual risk reduction of severe COVID-19		NNT to avert severe COVID-19 case[a]
			Absolute risk averted (cases per 100,000)	Relative risk averted (%)	
One-time booster[b]					
18-49 years	1,900	95 (83-125)	--	--	--
50-64 years	3,964	198 (180-236)	--	--	--
65-74 years	10,299	515 (487-557)	--	--	--
75+ years	27,598	1,380 (1,308-1,475)	--	--	--
Immunocompromised (Mild)	25,596	1,280 (1,194-1,394)	--	--	--
Immunocompromised (Moderate/Severe)	26,941	1,347 (1,246-1,481)	--	--	--
Annual booster					
18-49 years	1,669	83 (72-108)	12	12%	4,330
50-64 years	3,408	170 (156-204)	28	14%	1,799
65-74 years	8,900	445 (421-479)	70	14%	715
75+ years	23,892	1,195 (1,137-1,274)	185	13%	270
Immunocompromised (Mild)	23,608	1,180 (1,089-1,318)	99	8%	504
Immunocompromised (Moderate/Severe)	23,686	1,184 (1,090-1,310)	163	12%	308
Semiannual booster (every 6 months)					
18-49 years	1,447	72 (63-94)	23	24%	2,208
50-64 years	2,997	150 (137-176)	48	24%	1,035
65-74 years	7,752	388 (368-417)	127	25%	393
75+ years	21,026	1,051 (1,001-1,114)	329	24%	153
Immunocompromised (Mild)	22,100	1,105 (1,001-1,264)	175	14%	287
Immunocompromised (Moderate/Severe)	21,467	1,073 (982-1,199)	274	20%	183

[a]Estimated over 2-year simulation period in population of 1 million persons.
[b]One-time booster is the baseline intervention for risk reduction calculations.

Table S24: Sensitivity analysis of higher sub-clinical infection on the number of severe COVID-19 cases, risk, and number needed to treat to avert severe COVID-19 in six risk groups with different frequencies of COVID-19 booster vaccination.

	Total severe COVID-19 cases[a]	Absolute annual risk of severe COVID-19 (cases per 100,000; UI)	Annual risk reduction of severe COVID-19		NNT to avert severe COVID-19 case[a]
			Absolute risk averted (cases per 100,000)	Relative risk averted (%)	
One-time booster[b]					
18-49 years	1,657	83 (69-119)	--	--	--
50-64 years	3,444	172 (151-220)	--	--	--
65-74 years	9,247	462 (430-515)	--	--	--
75+ years	24,326	1,216 (1,128-1,356)	--	--	--
Immunocompromised (Mild)	23,654	1,183 (1,081-1,328)	--	--	--
Immunocompromised (Moderate/Severe)	25,118	1,256 (1,133-1,423)	--	--	--
Annual booster					
18-49 years	1,481	74 (61-104)	9	11%	5,682
50-64 years	3,020	151 (135-190)	21	12%	2,359
65-74 years	8,065	403 (443-376)	59	13%	847
75+ years	21,400	1,070 (1,001-1,178)	146	12%	342
Immunocompromised (Mild)	22,022	1,101 (990-1,268)	82	7%	613
Immunocompromised (Moderate/Severe)	22,161	1,108 (997-1,262)	148	12%	339
Semiannual booster (every 6 months)					
18-49 years	1,288	64 (54-89)	18	22%	2,711
50-64 years	2,653	133 (119-164)	40	23%	1,265
65-74 years	7,030	352 (329-385)	111	24%	452
75+ years	18,752	938 (883-1,023)	279	23%	180
Immunocompromised (Mild)	20,657	1,033 (912-1,217)	150	13%	334
Immunocompromised (Moderate/Severe)	20,018	1,001 (896-1,147)	255	20%	197

[a]Estimated over 2-year simulation period in population of 1 million persons.
[b]One-time booster is the baseline intervention for risk reduction calculations.

Table S25: Sensitivity analysis of lower vaccine effectiveness for subsequent doses after the first dose on the number of severe COVID-19 cases, risk, and number needed to treat to avert severe COVID-19 in six risk groups with different frequencies of COVID-19 booster vaccination.

	Total severe COVID-19 cases[a]	Absolute annual risk of severe COVID-19 (cases per 100,000; UI)	Annual risk reduction of severe COVID-19		NNT to avert severe COVID-19 case[a]
			Absolute risk averted (cases per 100,000)	Relative risk averted (%)	
One-time booster[b]					
18-49 years	1,949	97 (85-126)	--	--	--
50-64 years	4,041	202 (183-240)	--	--	--
65-74 years	10,422	521 (494-566)	--	--	--
75+ years	27,937	1,397 (1,329-1,504)	--	--	--
Immunocompromised (Mild)	25,797	1,290 (1,203-1,405)	--	--	--
Immunocompromised (Moderate/Severe)	27,340	1,367 (1,263-1,504)	--	--	--
Annual booster					
18-49 years	1,767	88 (77-116)	9	9%	5,495
50-64 years	3,587	179 (166-217)	23	11%	2,203
65-74 years	9,467	473 (448-509)	48	9%	1,048
75+ years	25,398	1,270 (1,210-1,358)	127	9%	394
Immunocompromised (Mild)	24,408	1,220 (1,131-1,347)	69	5%	720
Immunocompromised (Moderate/Severe)	24,832	1,242 (1,147-1,369)	125	9%	399
Semiannual booster (every 6 months)					
18-49 years	1,655	83 (72-108)	15	15%	3,402
50-64 years	3,378	169 (154-201)	33	16%	1,509
65-74 years	8,843	442 (417-472)	79	15%	634
75+ years	23,672	1,184 (1,126-1,261)	213	15%	235
Immunocompromised (Mild)	23,475	1,174 (1,078-1,314)	116	9%	431
Immunocompromised (Moderate/Severe)	23,306	1,165 (1,073-1,291)	202	15%	248

[a]Estimated over 2-year simulation period in population of 1 million persons.
[b]One-time booster is the baseline intervention for risk reduction calculations.

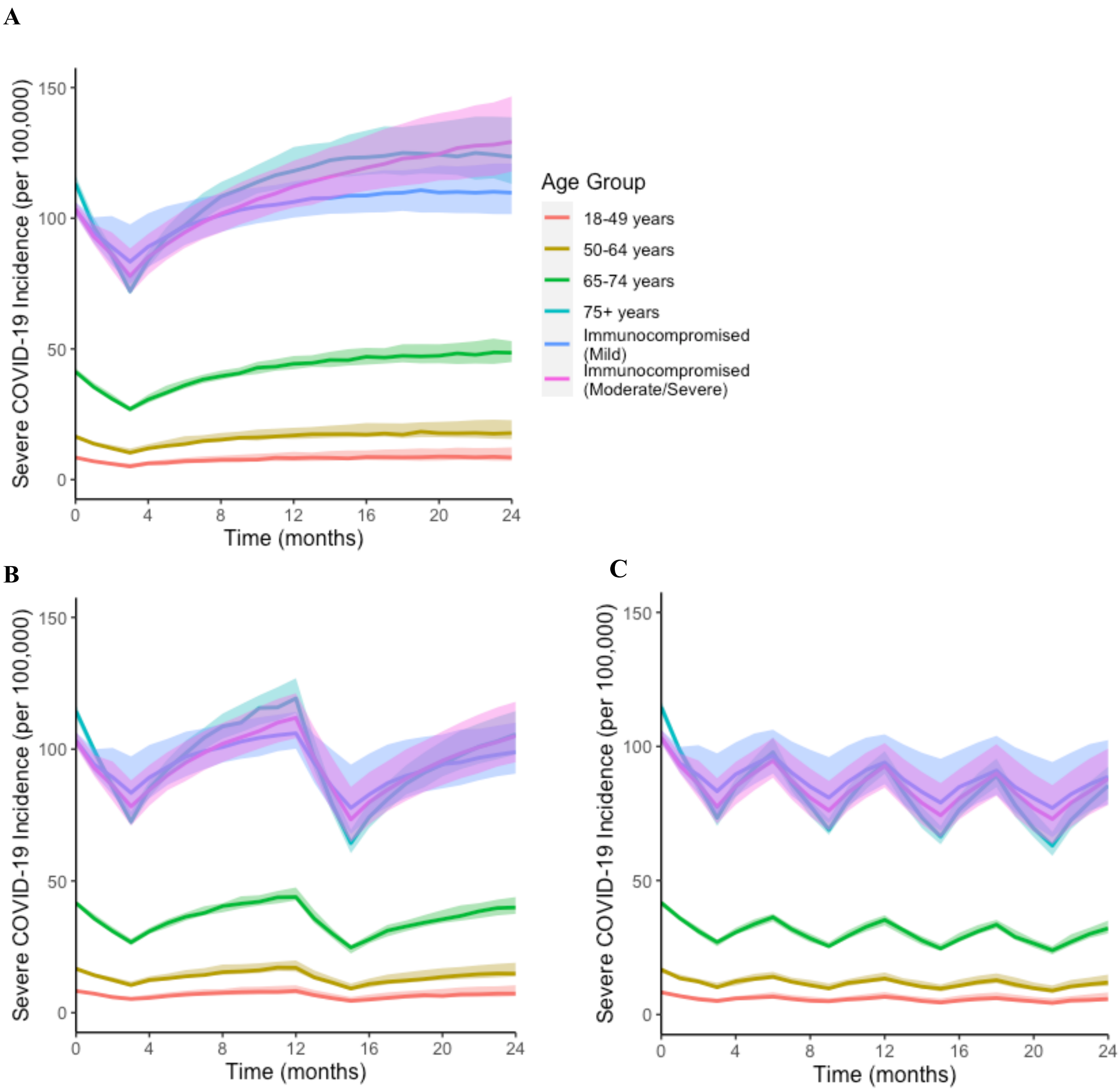

Figure S10: Monthly incidence of severe COVID-19 in four age groups and two immunocompromised groups over a two-year simulation period with different frequencies of COVID-19 booster vaccination. We simulated three COVID-19 booster vaccine schedules with the mRNA dose: (A) One-time booster (total of 1 dose); (B) annual booster (total of 2 doses); (C) booster every 6 months (total of 4 doses). We estimated incidence of severe COVID-19 per 100,000 persons (y-axis) over time in months (x-axis), by age group and immunocompromised population in Panels A-C. We modeled the protection of a booster (administered in the population over a 3-month period) to restore vaccine-induced protection that waned over time based on published literature, which reduced severe COVID-19 cases. More frequent booster vaccination (panel B-C) reduced total severe COVID-19 cases compared to one-time booster (panel A), and this benefit was most pronounced in the oldest age groups. The uncertainty intervals are based on uncertainty in waning data, in addition to uncertainty in baseline seroprevalence and non-severe infection multiplier estimates.

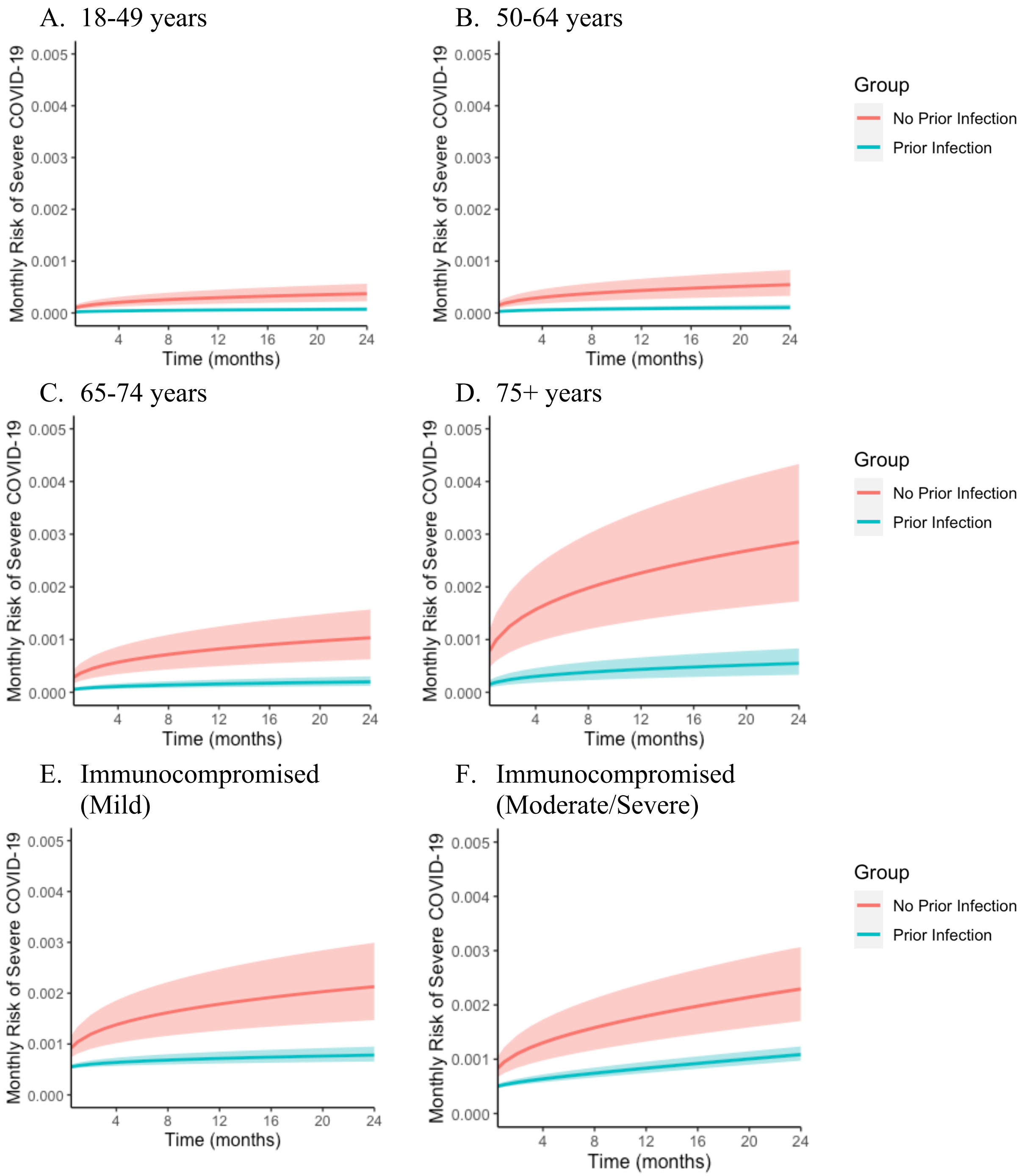

Figure S11: Risk of severe COVID-19 over time by baseline risk and waning protection. We modeled risk of severe COVID-19 by baseline risk and time since last immune event (vaccine or infection) by multiplying age-specific lambdas to protection estimates over time. The risk groups modeled here are the (A) 18-49 years; (B) 50-64 years; (C) 65-74 years; (D) 75+ years; (E) Immunocompromised (mild); and (F) Immunocompromised (moderate/severe).

A. 18-49 years

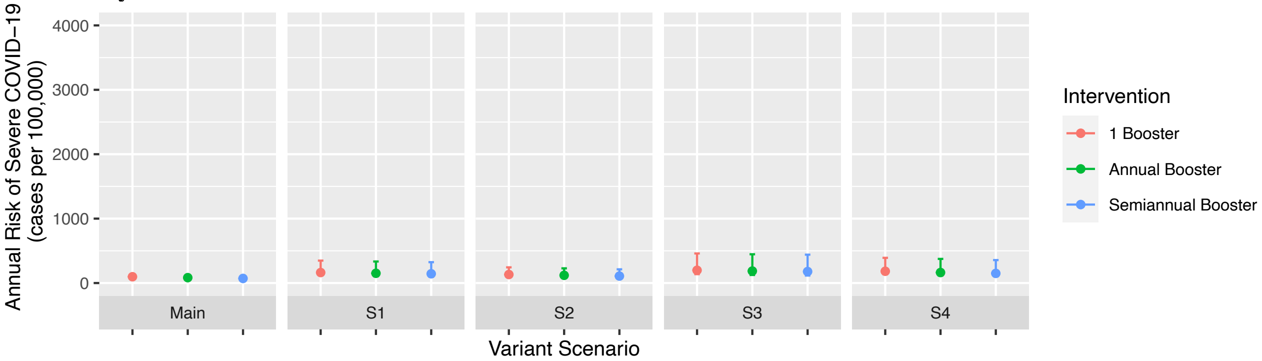

B. 50-64 years

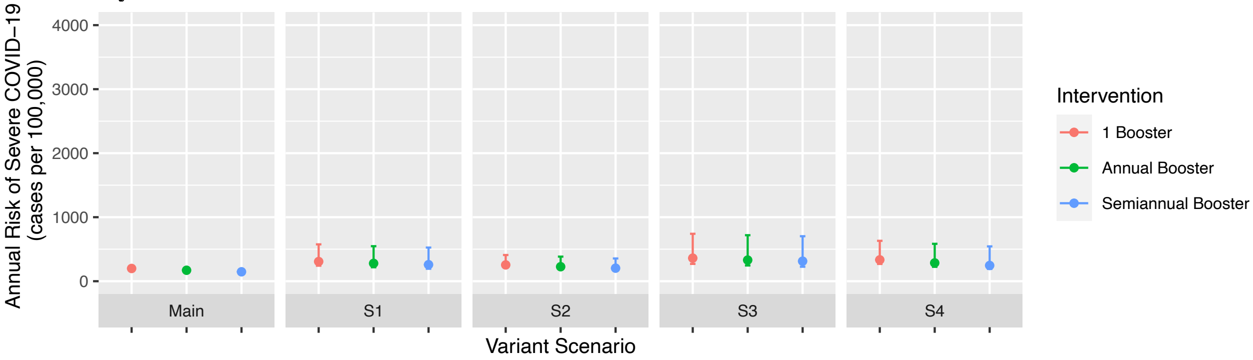

C. 65-74 years

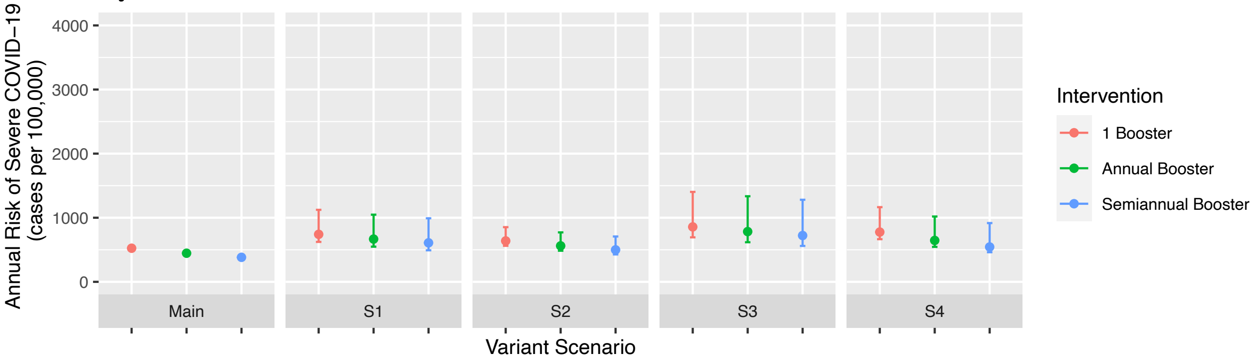

D. 75+ years

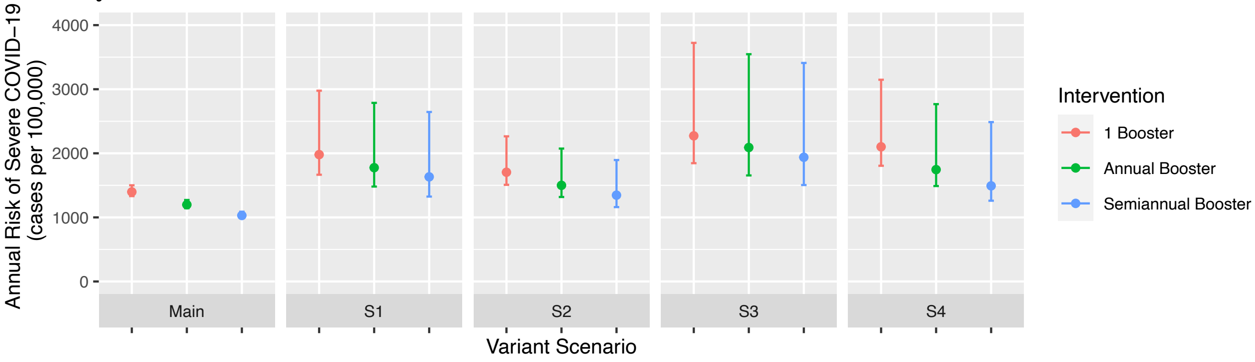

E. Immunocompromised (Mild)

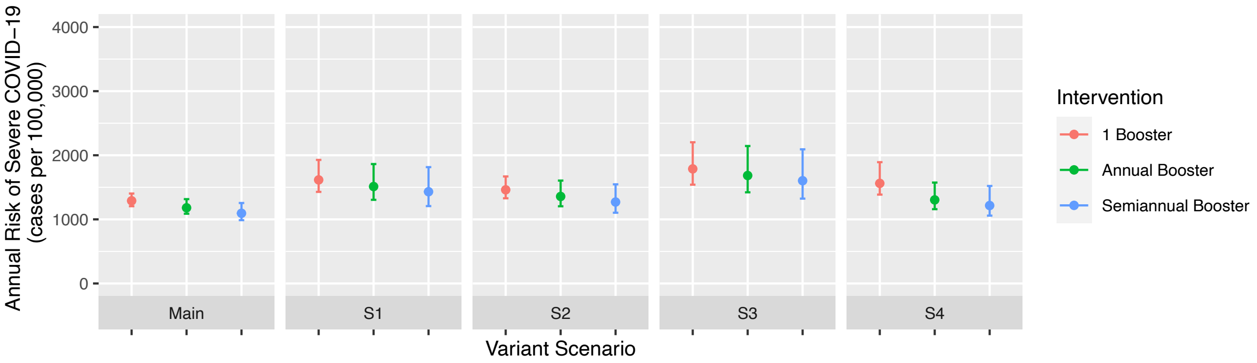

F. Immunocompromised (Moderate/Severe)

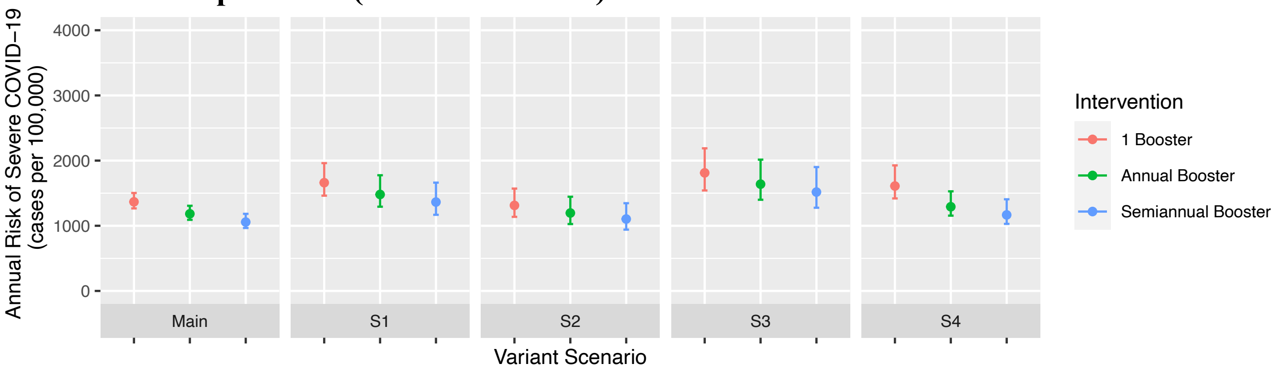

Figure S12: Scenario analysis on emergence of novel SARS-CoV-2 variants with immune evasion (10% reduction in immunity) comparing severe COVID-19 risk with different frequencies of COVID-19 booster vaccination. We simulated four scenarios on emergence of novel variant(s) with reduced susceptibility to protection generated by prior vaccination and natural infection. Under each variant scenario analysis, we simulated three frequencies of COVID-19 booster vaccine for four age groups and two immunocompromised groups. We plotted absolute annual risk of severe COVID-19 over a two-year simulation. The vertical bars represent uncertainty intervals and capture the full range of varied model parameters (n=25 simulations per model parameter set), while the point estimate uses base case assumptions of model inputs. Intervals are designed to demonstrate uncertainty within a single vaccine strategy; comparison between vaccine strategies should use the same assumed baseline conditions.

A. **18-49 years**

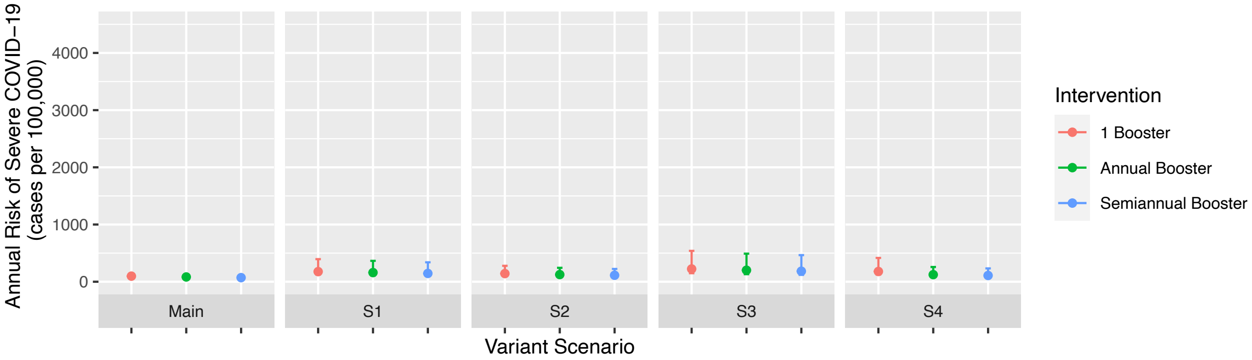

B. **50-64 years**

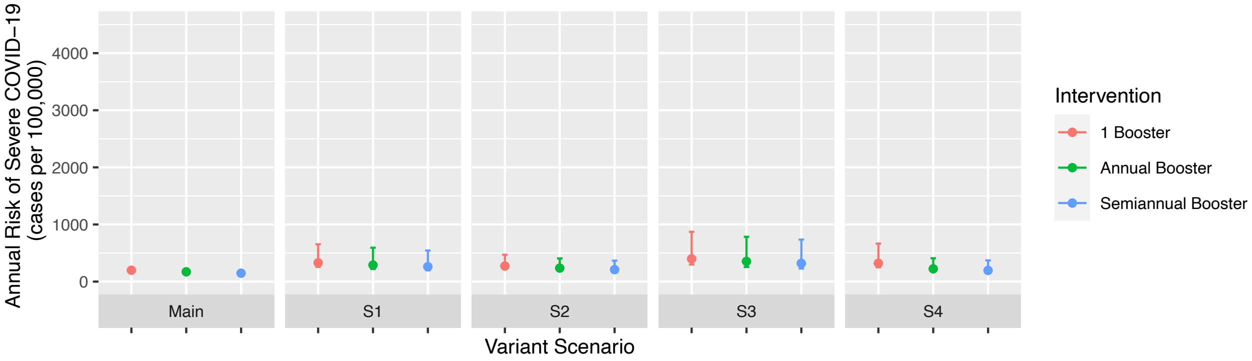

C. **65-74 years**

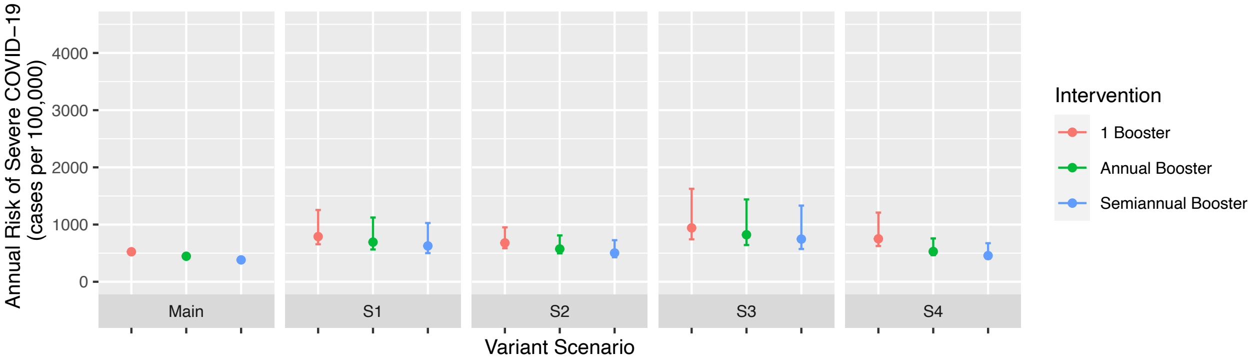

D. **75+ years**

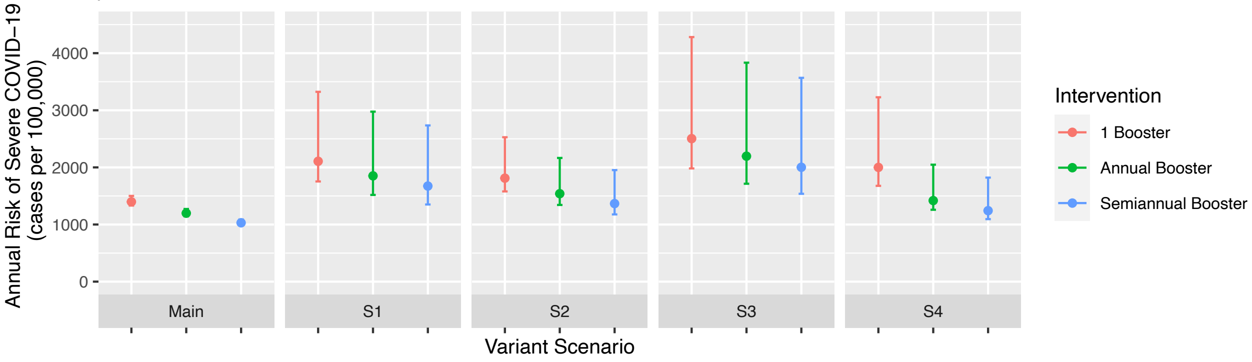

E. **Immunocompromised (Mild)**

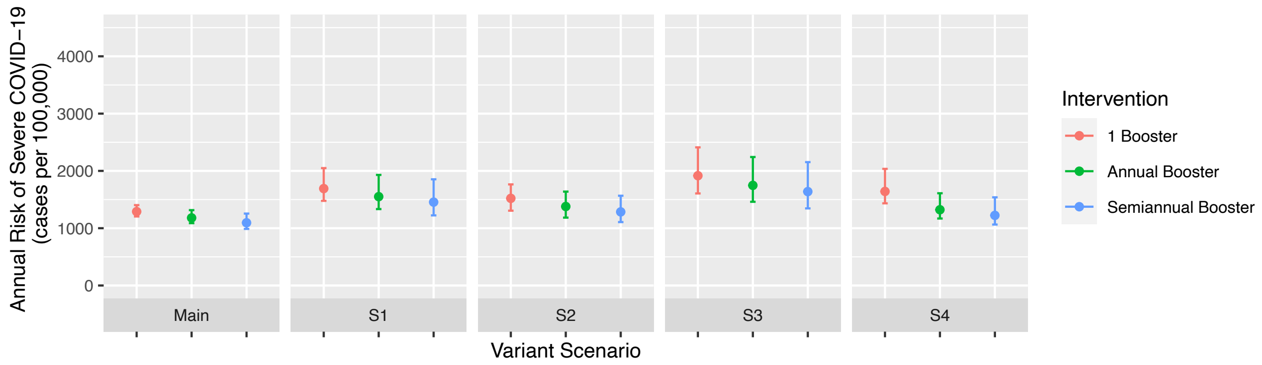

F. Immunocompromised (Moderate/Severe)

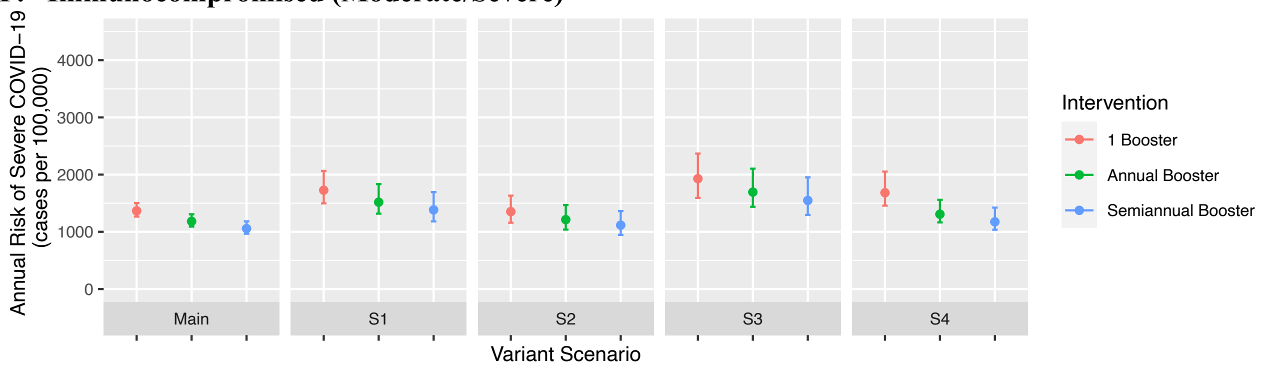

Figure S13: Scenario analysis on emergence of novel SARS-CoV-2 variants with immune evasion (10% absolute reduction and 5% increased rate of waning) comparing severe COVID-19 risk with different frequencies of COVID-19 booster vaccination. We simulated four scenarios on emergence of novel variant(s) with reduced susceptibility to protection generated by prior vaccination and natural infection. Under each variant scenario analysis, we simulated three frequencies of COVID-19 booster vaccine for four age groups and two immunocompromised groups. We plotted absolute annual risk of severe COVID-19 over a two-year simulation. The vertical bars represent uncertainty intervals and capture the full range of varied model parameters (n=25 simulations per model parameter set), while the point estimate uses base case assumptions of model inputs. Intervals are designed to demonstrate uncertainty within a single vaccine strategy; comparison between vaccine strategies should use the same assumed baseline conditions.

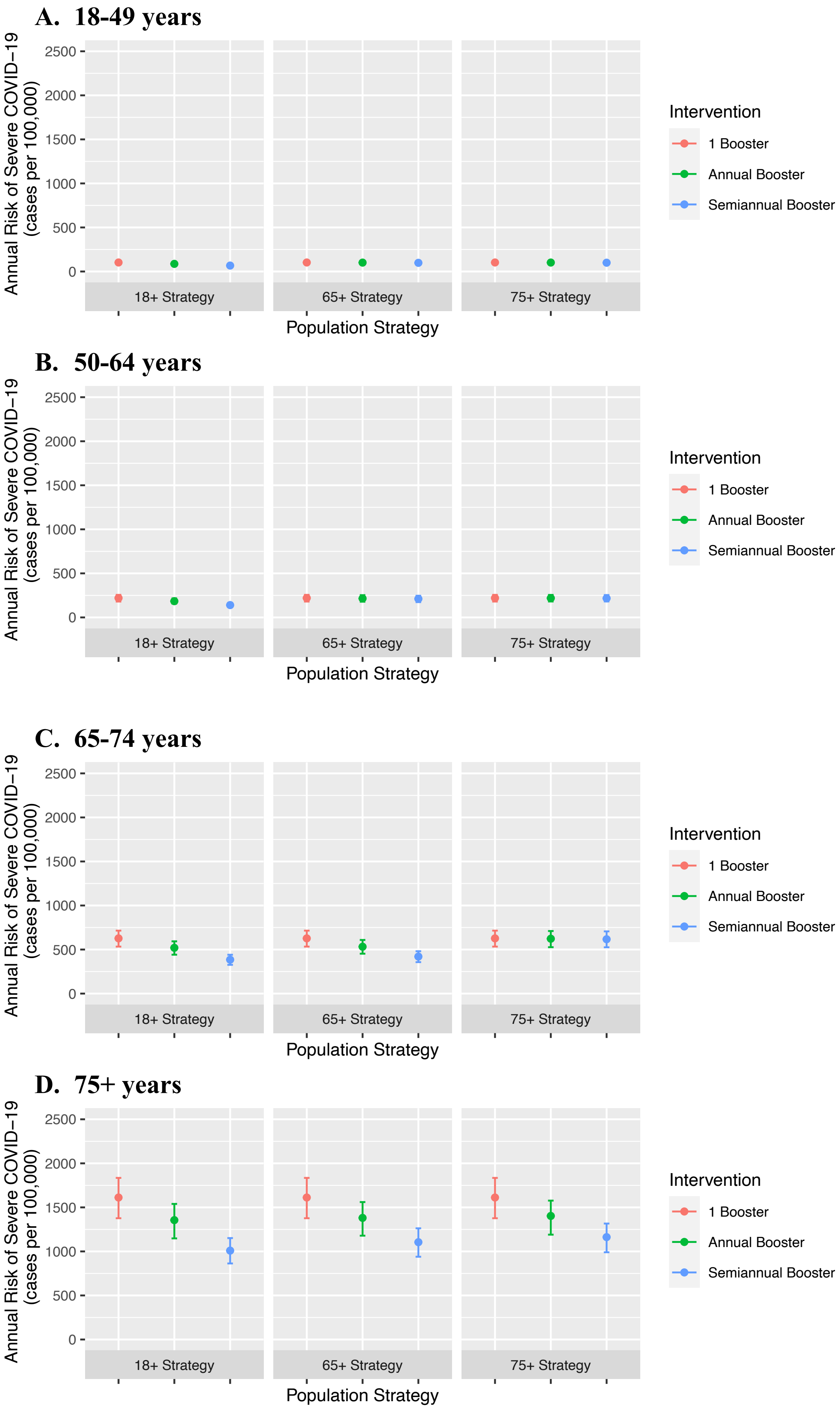
A. 18-49 years
B. 50-64 years
C. 65-74 years
D. 75+ years
Annual Risk of Severe COVID−19
(cases per 100,000)
2500
2000
1500
1000
500
0
18+ Strategy
65+ Strategy
75+ Strategy
Population Strategy
Intervention
1 Booster
Annual Booster
Semiannual Booster

E. Immunocompromised (Mild)

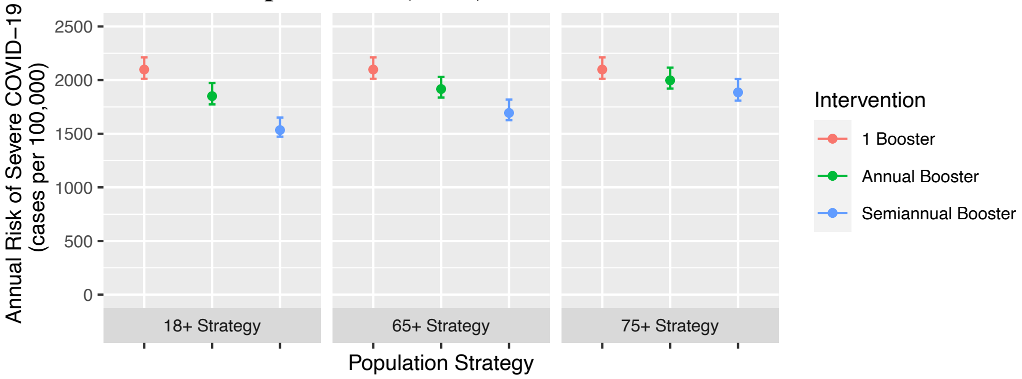

F. Immunocompromised (Moderate/Severe)

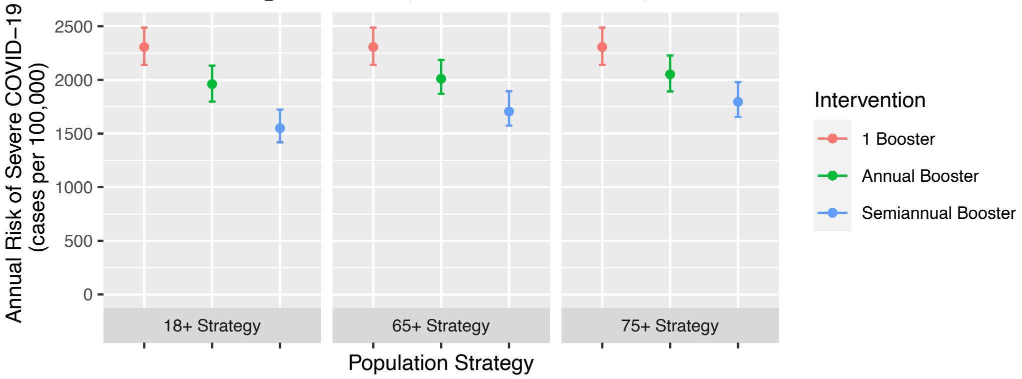

Figure S14: Scenario analysis using a dynamic transmission model under realistic coverage assumptions to estimate the impact of indirect effects on COVID-19 booster vaccination strategies in four age groups and two immunocompromised groups. We used a dynamic transmission model to compare different frequencies of COVID-19 booster vaccine in the following groups: (A) 75+ years, moderate/severe immunocompromised group; (B) 65+ years and all immunocompromised groups; and (C) 18+ years in all groups. We assumed a background of one-time booster vaccination at the start of the simulation in adults (18+ years) with age-specific coverage based on current uptake. We plotted absolute annual risk of severe COVID-19 over a two-year simulation in four age groups and two immunocompromised groups, to compare the indirect effects of booster vaccination on all risk groups. Table S7 reports the coverage estimates (realistic vaccine uptake assumption). The vertical bars represent uncertainty intervals and capture the full range of varied model parameters (n=25 simulations per model parameter set), while the point estimate uses base case assumptions of model inputs. Intervals are designed to demonstrate uncertainty within a single vaccine strategy; comparison between vaccine strategies should use the same assumed baseline conditions.

A. 18-49 years

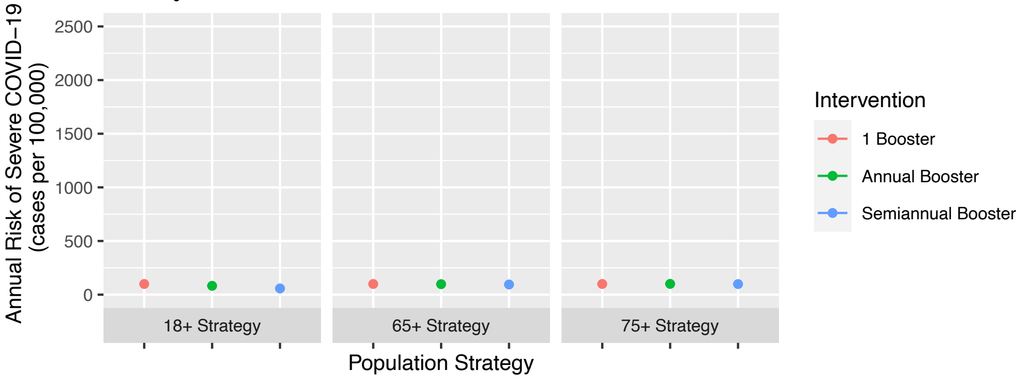

B. 50-64 years

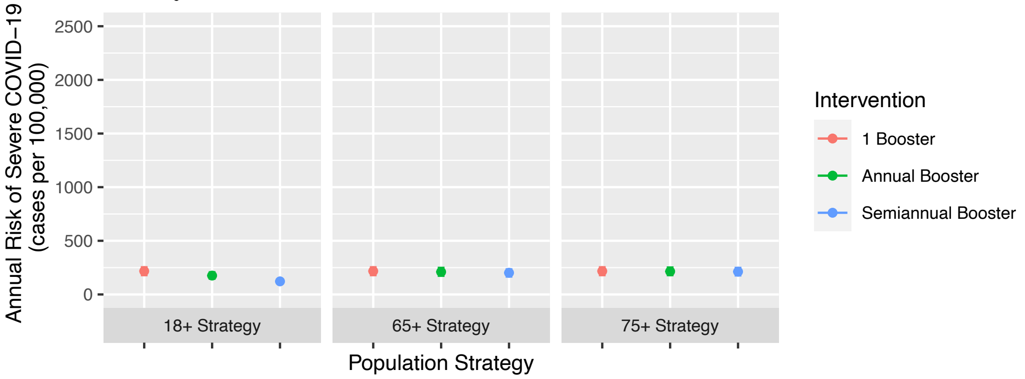

C. 65-74 years

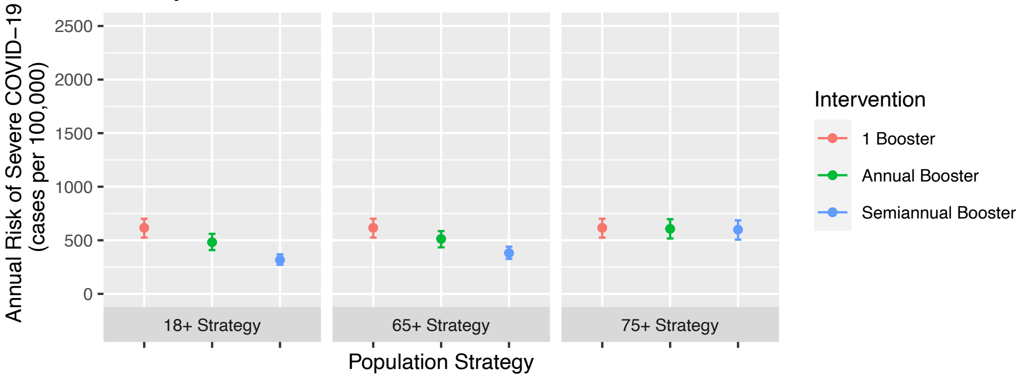

D. 75+ years

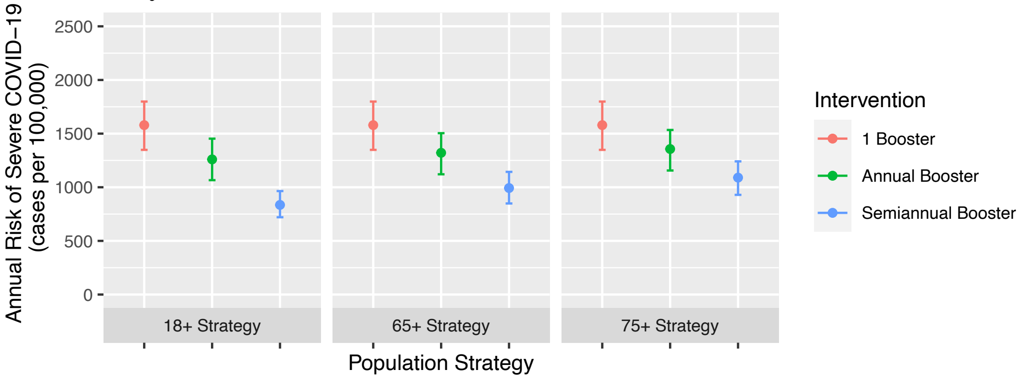

E. Immunocompromised (Mild)

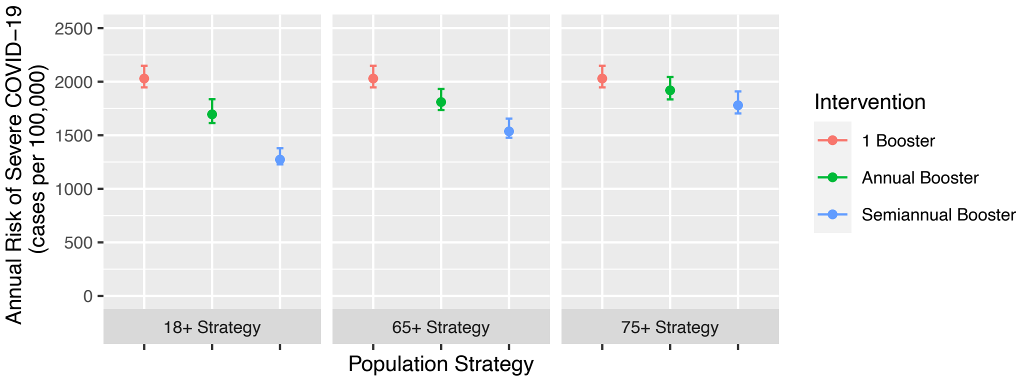

F. Immunocompromised (Moderate/Severe)

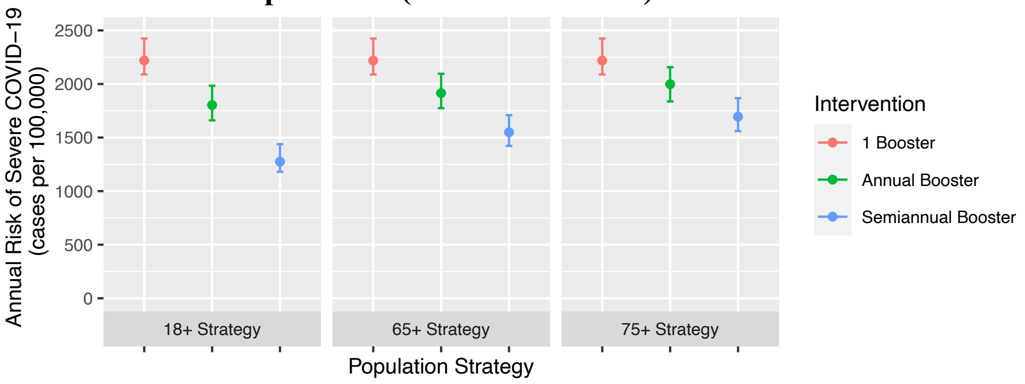

Figure S15: Scenario analysis using a dynamic transmission model under optimistic coverage assumptions to estimate the impact of indirect effects on COVID-19 booster vaccination strategies in four age groups and two immunocompromised groups. We used a dynamic transmission model to compare different frequencies of COVID-19 booster vaccine in the following groups: (A) 75+ years, moderate/severe immunocompromised group; (B) 65+ years and all immunocompromised groups; and (C) 18+ years in all groups. We assumed a background of one-time booster vaccination at the start of the simulation in adults (18+ years) with age-specific coverage based on optimistic uptake assumptions. We plotted absolute annual risk of severe COVID-19 over a two-year simulation in four age groups and two immunocompromised groups, to compare the indirect effects of booster vaccination on all risk groups. Table S7 reports the coverage estimates (optimistic vaccine uptake assumption). The vertical bars represent uncertainty intervals and capture the full range of varied model parameters (n=25 simulations per model parameter set), while the point estimate uses base case assumptions of model inputs. Intervals are designed to demonstrate uncertainty within a single vaccine strategy; comparison between vaccine strategies should use the same assumed baseline conditions.
