# Supplementary material for: Comparing frequency of booster vaccination to prevent severe COVID-19 by risk group in the United States
Source: Nat Commun. 2024 Mar 6;15:1883. doi: 10.1038/s41467-024-45549-9 (PMC10917753; doi:10.1038/s41467-024-45549-9)
Supplement: Supplementary file 3 — Reporting Summary [file 41467_2024_45549_MOESM3_ESM.pdf]

nature portfolio

Corresponding author(s): Nathan Lo

Last updated by author(s): Jan 19, 2024

Reporting Summary

Nature Portfolio wishes to improve the reproducibility of the work that we publish. This form provides structure for consistency and transparency in reporting. For further information on Nature Portfolio policies, see our Editorial Policies and the Editorial Policy Checklist.

Statistics

For all statistical analyses, confirm that the following items are present in the figure legend, table legend, main text, or Methods section.

n/a	Confirmed	
☐	☒	The exact sample size (*n*) for each experimental group/condition, given as a discrete number and unit of measurement
☒	☐	A statement on whether measurements were taken from distinct samples or whether the same sample was measured repeatedly
☒	☐	The statistical test(s) used AND whether they are one- or two-sided<br>*Only common tests should be described solely by name; describe more complex techniques in the Methods section.*
☐	☒	A description of all covariates tested
☐	☒	A description of any assumptions or corrections, such as tests of normality and adjustment for multiple comparisons
☐	☒	A full description of the statistical parameters including central tendency (e.g. means) or other basic estimates (e.g. regression coefficient) AND variation (e.g. standard deviation) or associated estimates of uncertainty (e.g. confidence intervals)
☒	☐	For null hypothesis testing, the test statistic (e.g. *F*, *t*, *r*) with confidence intervals, effect sizes, degrees of freedom and *P* value noted<br>*Give P values as exact values whenever suitable.*
☒	☐	For Bayesian analysis, information on the choice of priors and Markov chain Monte Carlo settings
☒	☐	For hierarchical and complex designs, identification of the appropriate level for tests and full reporting of outcomes
☒	☐	Estimates of effect sizes (e.g. Cohen's *d*, Pearson's *r*), indicating how they were calculated

Our web collection on statistics for biologists contains articles on many of the points above.

Software and code

Policy information about availability of computer code

Data collection	No software was used for data collection.
Data analysis	R statistical software (version 4.1.2), R packages include 'tidyverse', 'reshape2', 'lubridate', 'scales', 'lme4', 'data.table', 'foreach', 'doParallel', and 'here'. Code is made publicly available via Github (https://github.com/hailey-park/booster-timing).

For manuscripts utilizing custom algorithms or software that are central to the research but not yet described in published literature, software must be made available to editors and reviewers. We strongly encourage code deposition in a community repository (e.g. GitHub). See the Nature Portfolio guidelines for submitting code & software for further information.

Data

Policy information about availability of data

All manuscripts must include a data availability statement. This statement should provide the following information, where applicable:

- Accession codes, unique identifiers, or web links for publicly available datasets
- A description of any restrictions on data availability
- For clinical datasets or third party data, please ensure that the statement adheres to our policy

This study used publicly available secondary datasets from the US Centers for Disease Control and Prevention. This includes COVID-19 hospitalization data from the COVID-NET surveillance system (link: https://data.cdc.gov/Public-Health-Surveillance/Weekly-Rates-of-Laboratory-Confirmed-COVID-19-Hosp/twtx-bfcw/about_data), rates of COVID-19 cases and deaths by vaccination status (https://data.cdc.gov/Public-Health-Surveillance/Rates-of-COVID-19-Cases-or-Deaths-by-

Age-Group-and/d6p8-wqjm/about_data), and age-specific seroprevalence estimates (https://covid.cdc.gov/covid-data-tracker/#national-lab). More details on these datasets can be found in the Appendix. Input data and processing scripts can be found at https://github.com/hailey-park/booster-timing. Analyses were conducted in R (version 4.1.2).

Research involving human participants, their data, or biological material

Policy information about studies with human participants or human data. See also policy information about sex, gender (identity/presentation), and sexual orientation and race, ethnicity and racism.

Reporting on sex and gender	This simulated population did not include sex or gender, given limited data in differences in vaccine effectiveness and waning. All sexes are therefore represented in this study.
Reporting on race, ethnicity, or other socially relevant groupings	This simulated population did not include race or ethnicity.
Population characteristics	We analyzed detailed COVID-19 surveillance data and seroprevalence estimates from US Centers for Disease Control and Prevention, which represented the entire United States population. We estimated severe COVID-19 incidence and seroprevalence by age groups, including 18-49 years, 50-64 years, 65-74 years, and 75+ years, and an immunocompromised population.
Recruitment	N/A
Ethics oversight	This study is not human subjects research given this is a computer simulation study using publicly available, secondary datasets with aggregated estimates that are not identifiable, which was then used to create a simulation study.

Note that full information on the approval of the study protocol must also be provided in the manuscript.

Field-specific reporting

Please select the one below that is the best fit for your research. If you are not sure, read the appropriate sections before making your selection.

☒ Life sciences ☐ Behavioural & social sciences ☐ Ecological, evolutionary & environmental sciences

For a reference copy of the document with all sections, see nature.com/documents/nr-reporting-summary-flat.pdf

Life sciences study design

All studies must disclose on these points even when the disclosure is negative.

Sample size	No formal sample size calculation was performed for this study. The input data for the simulation model included all reported cases of severe COVID-19 in the United States, and we used all available case data. The simulation model itself chose a population size of 1 million, which was designed to represent a large county in the United States, and also provided stability of COVID-19 outcome estimates.
Data exclusions	COVID-19 outcomes from children (<18 years) were excluded, given this study focused on the adult population.
Replication	Analysis was conducted with automated code scripts, and all code was reviewed by another co-author.
Randomization	N/A; We set a seed so that all randomized processes can be reproducible.
Blinding	N/A; Blinding is not applicable to this simulation study.

Reporting for specific materials, systems and methods

We require information from authors about some types of materials, experimental systems and methods used in many studies. Here, indicate whether each material, system or method listed is relevant to your study. If you are not sure if a list item applies to your research, read the appropriate section before selecting a response.

Materials & experimental systems

n/a	Involved in the study	
☒	☐	Antibodies
☒	☐	Eukaryotic cell lines
☒	☐	Palaeontology and archaeology
☒	☐	Animals and other organisms
☐	☒	Clinical data
☒	☐	Dual use research of concern
☒	☐	Plants

Methods

n/a	Involved in the study	
☒	☐	ChIP-seq
☒	☐	Flow cytometry
☒	☐	MRI-based neuroimaging

Clinical data

Policy information about clinical studies

All manuscripts should comply with the ICMJE guidelines for publication of clinical research and a completed CONSORT checklist must be included with all submissions.

Clinical trial registration: N/A

Study protocol: N/A

Data collection: We analyzed COVID-19 surveillance data and seroprevalence estimates from US Centers for Disease Control and Prevention, which represented the entire United States population. Incidence estimates for severe COVID-19 (defined by hospitalization or death) were generated using publicly available US CDC data, averaging over 6 months preceding bivalent vaccination (March 2022-August 2022). Age-specific seroprevalence estimates were based on the most recent survey from February 2022, with additional adjustment based on reported case data to revise these estimates to reflect the seroprevalence as of September 2022 (see Appendix).

Outcomes: Primary outcome is severe COVID-19, defined as COVID-19-related hospitalization or death. The attribution of COVID-19 to these outcomes were based on provider reporting on hospitalization records and death certificates.

Plants

Seed stocks: N/A

Novel plant genotypes: N/A

Authentication: N/A
